# Mining viruses in public databases unveils the diversity within the *Deltaflexiviridae* family

**DOI:** 10.1007/s00705-026-06648-8

**Published:** 2026-06-26

**Authors:** Jhon Eric Alves Fernandes, Tatsuya Nagata, Fernando Lucas Melo

**Affiliations:** 1 Programa de Pós Graduação Biologia Microbiana, Universidade de Brasília, Brasilia, Brazil; 2 OnSite Genomics, Brasilia, Brazil; 3 Empresa Brasileira de Pesquisa Agropecuária (Embrapa), Brasilia, Brazil

## Abstract

Cloud computing platforms aided the scalability and applicability of viral mining in genomic databases. The Serratus project reported SRA accessions that may contain viral sequences. This study analyzed SRA accessions containing sequences similar to *Tymovirales* members to verify the presence of sequence data derived from viral genomes. All steps in the genome mining analysis were performed by a pipeline running on virtual machines hosted on the Google Cloud Platform. Manual curation of the pipeline output discovered 111 putative genomes in the analyzed SRAs. Among the genomes identified, four were classified as isolates within the *Betaflexiviridae* family, and two were putative new members of the *Alphaflexiviridae* family. Another four sequences were likely classified in a family not yet accepted by the ICTV. The phylogenetic reconstruction of the *Deltaflexiviridae* family revealed three distinct clades, one of them containing 81 genomes (34 putative new species), a second clade with 18 novel genomes (7 putative new species), and a third with one putative new species. Given the high divergence between these three groups, we suggest the establishment of a new family, “*Epsilonflexiviridae”*, and the split of the *Deltaflexiviridae* family into two by establishing the family “*Zetaflexiviridae”*. The results of this work provide insight into important aspects of the evolutionary history of the order *Tymovirales* and offer new ways for virus-mining projects in genomic databases.

**Supplementary Information:**

The online version contains supplementary material available at 10.1007/s00705-026-06648-8.

## Introduction

The rapid growth of the Short Read Archive (SRA), a database that stores raw data from genomic sequencing experiments [1], sparked interest in using this information to survey new virus-derived data [2, 3]. Such efforts contributed to expanding the diversity boundaries of plant viruses [4–7] and viroids [8], with significant economic interest, and helped unveil the diversity within groups of previously understudied viruses [7, 9–11]. However, the sheer volume of information poses data-processing challenges. Nevertheless, cloud computing tools can overcome these hurdles by providing commercially available massively distributed computational resources [12]. The mining process requires multiple intensive, repetitive computational tasks, necessitating automated data processing. These steps are often sequential, with the output of one program step fed into the next, facilitating their organization into automated computational pipelines [13]. The construction of genomic pipelines is an area of intense research, and several pipelines are available for virus discovery [14–18]. Although extremely useful and easy to use, it is often very challenging to modify the available automated pipelines for the use of applications outside their intended scope. A possible solution is to use pipeline platforms that offer flexibility and reproducibility [19, 20], combined with straightforward integration with cloud computing resources [19]. These platforms often have a very active community of collaborators who share their reproducible pipelines for general use, combining flexibility with a less steep learning curve [21]. Still, the sheer volume of data is an obstacle in itself. However, this barrier has also been reduced by the recently described Serratus project [22]. Using an efficient cloud computing infrastructure on Amazon Web Services (AWS), the authors successfully pre-aligned numerous accessions and identified those likely to contain virus sequences [22].

The family *Deltaflexiviridae* is classified in the *Tymovirales* order, and comprises RNA viruses with a genome length of approximately 8 kb and a poly(A) tail at the 3’ end [23]. Genome organization varies considerably. All viruses have a polyprotein gene, but other genes can be different. These additional ORFs, whose functions have not yet been elucidated, are all annotated using a consistent convention. A “HP” prefix, which stands for hypothetical protein, is appended by a number (HP2, HP3, HP4, and HP5).

Using the Serratus resource, we investigated candidate accessions that may harbor viral genomes from the family *Deltaflexiviridae*. Our findings reveal tens of possible new viruses and shed light on the evolution of the order *Tymovirales* as a whole.

## Methods

### Assembly pipeline

**Fig. 1:**
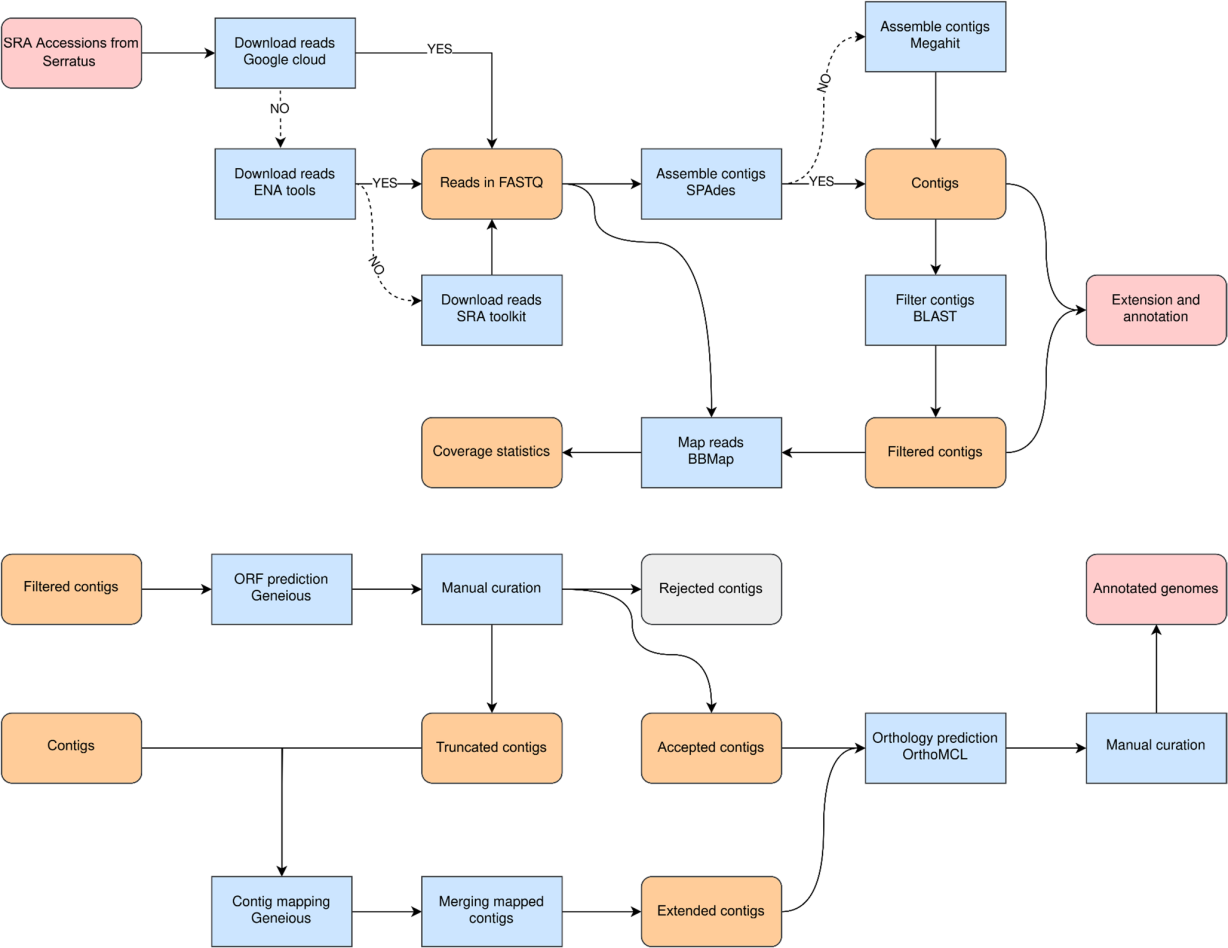
Flow chart of the pipeline steps. The top part of the pipeline refers to the assembly steps, which were executed on a virtual machine on google cloud with 200 GB of storage, 32 threads, and 64 GB of RAM. This portion of the pipeline takes an SRA accession as input and returns two sets of assembled contigs. The first set contains contigs at least 500 bp long, which will be used to extend and refine putative viral genomes. The other set comprises contigs filtered by BLAST similarity to known viruses. The bottom part refers to the extension and annotation steps, which are executed locally. This portion of the pipeline is not fully automated, since manual curation is required

The mined viral sequences described in this work were obtained using an automated pipeline running on Google Cloud servers that takes a list of SRA accessions (Supplementary Table 1) as input and returns probable viral contigs and coverage statistics (Fig. 1). The accessions with likely viral sequences were downloaded from the Serratus interface, which provides a list of SRA accessions separated by family. For this work, 204 SRA accessions similar to deltaflexivirids were downloaded and used as input for the pipeline. The pipeline was executed on a virtual machine on Google Cloud with 200 GB of storage, 32 threads, and 64 GB of RAM.

The first pipeline step is to download the raw reads from the SRA database, which is available via Google Cloud servers. When available, the raw reads were simply copied from the cloud, eliminating the need for data transfer. When this was not possible, the reads were acquired using the ENA toolkit because it provided faster download speeds. Accessions unavailable through the ENA database [24] were downloaded using the SRA toolkit. Metadata associated with the SRA accession were obtained via EUtilities [25]. SPAdes was used to assemble the downloaded reads, with the rnaviralspades option [26] enabled and all other options set to their default values. When SPAdes returned errors, Megahit was used with default settings [27] (Fig. 1). Accessions for which neither assembler could assemble the reads were discarded. Contigs shorter than 500 bp were discarded, and the remaining contigs were filtered. Given the focus on the *Deltaflexiviridae* family, the assembled contigs were filtered using BLAST [28] against a custom database containing amino acid sequences of viruses in this family deposited in public protein databases. After filtering, the raw reads were mapped to the filtered sequences using BBmap [29] to calculate coverage statistics.

### Manual curation and annotation

The virus and virus-like genomic sequences were manually curated in the Geneious software [30]. ORFinder was used to predict putative ORFs with ATG as the only start codon and a minimum ORF length of 150 nt. The curation process accounted for genome composition; that is, predicted ORFs were compared with those identified in available genomes. A genome was considered complete when all of its predicted ORFs contained both the start codon and the stop codon. An iterative extension protocol was then applied to genomes with interrupted ORFs or that were below the expected length for the group. The incomplete genomes were used as references for mapping against the unfiltered contigs using Geneious [30]. To extend the genome, the non-overlapping portion of the alignment was added to the reference only if the overlapping mapped contigs contained no more than two mutations. Viral sequences successfully extended using this method were remapped against the raw reads with BBmap [29], and coverage statistics were calculated. ORFs predicted with ORFinder built into Geneious were manually curated by removing internal and reverse coding frames. The ORF annotation step combined information from an OrthoMCL [31] orthologous search and InterProScan [32] conserved-domain predictions to curate the ORFs. Additional annotations of the clusters were assigned by manual searches using the web version of InterProScan [33]. The annotated polyprotein ORFs were then extracted for further analysis. Pairwise identities between polyprotein amino acid sequences were calculated using SDT [34] with Muscle [35] as the aligner. The phylogenetic reconstructions of the polyprotein sequences were estimated by the command-line program IQ-Tree version 1.6 [36], which includes a step to choose the best-fit substitution model. The sequences were aligned by MAFFT [37] and trimmed using the “automated1” option in TrimAL [38].

To elucidate the placement of the new viruses within this order, a dataset containing members of the order was used to construct the phylogeny of the group. This dataset combines reference genomes from the *Alphaflexiviridae*, *Betaflexiviridae*, *Deltaflexiviridae*, and *Tymoviridae* families with sequences obtained through an NCBI database search [25]. Additionally, a broader BLAST similarity search was performed against a database containing all proteins deposited in the Virus database at NCBI [39]. After filtering hits that generated shorter alignments, the corresponding genomes were also added to the dataset. The polyprotein amino acid sequences were then extracted, and the ones containing very short or truncated ORFs were excluded from the analysis. The online tool iTOL [40] was used to visualize the phylogenetic trees and genome organization. The summary figures were plotted using the Python package plotly.

## Results

### Pipeline efficiency

**Fig. 2:**
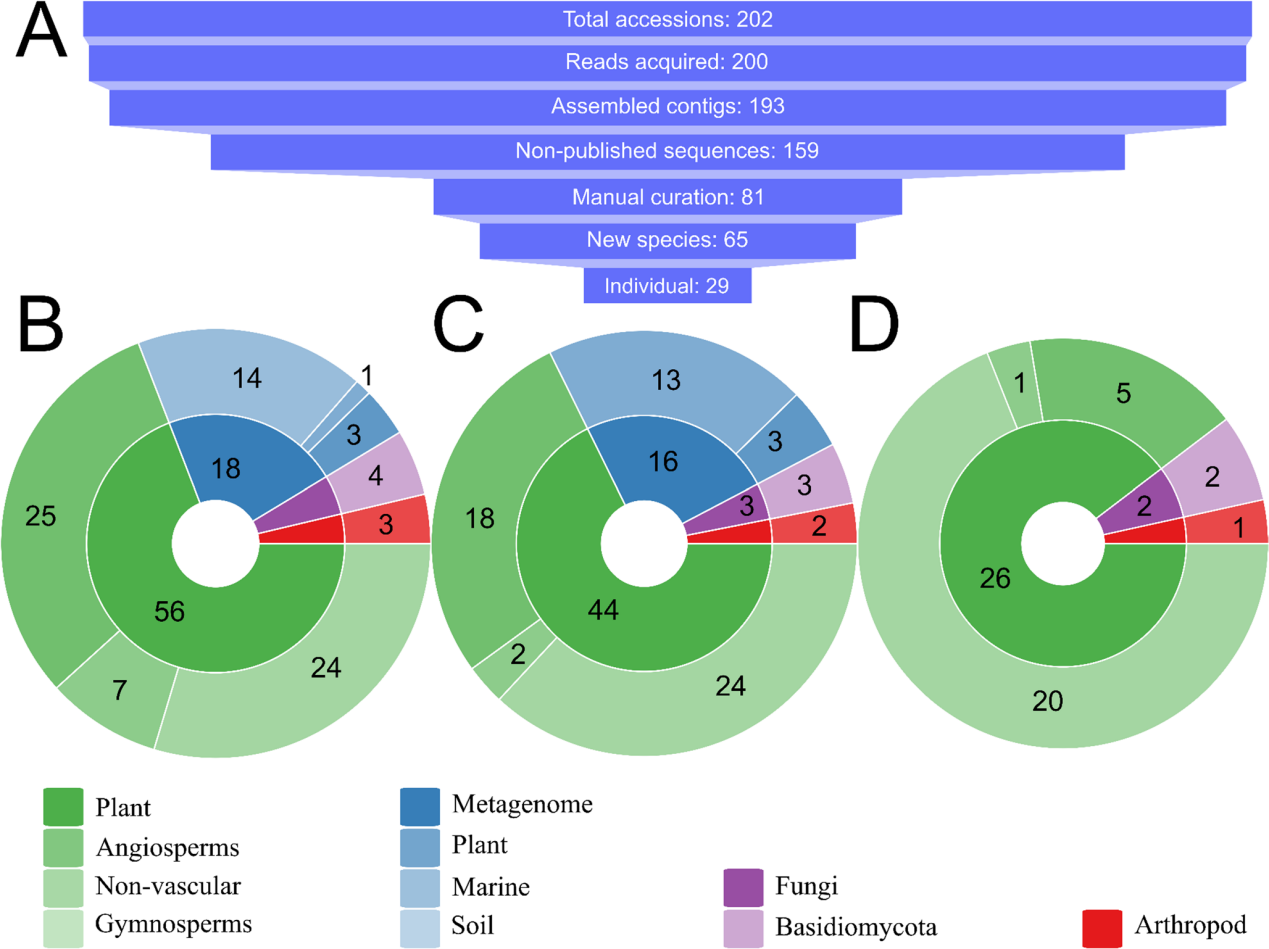
Pipeline efficiency by accession. (**A**) Funnel plot presenting the number of accessions that continued to the next step of the pipeline. (**B**) Source of the sample being sequenced in the accessions that had yielded a curated genome. (**C**) Source of the sequenced samples that yielded sequences with the polyprotein amino acid sequence sharing less than 90% identity to existing sequences. (**D**) Number of accessions in which the sequenced sample yielded a new species that originated from a single individual

The pipeline was run on 202 accessions available on the Serratus platform. Despite indications that all accessions contained viral sequences, our pipeline failed to detect new genomes in more than half of them. The reads from two accessions could not be retrieved. The pipeline yielded 193 contig sets likely of viral origin. Similarity searches revealed that 34 contig sets had been previously published and were discarded. Of the 159 remaining sets, only 81 passed the manual curation step (Fig. 2A).

Of the 81 SRA accessions with curated genomes, 56 originated from plant samples (Fig. 2B), of which 41 contained new species (Fig. 2C). However, only 26 plant accessions with putative new species originated from single-individual samples (Fig. 2D). Notably, the overrepresentation of samples containing new species was observed in Gymnosperms. A single sample from non-vascular plants was sequenced from single branches and leaves of the liverwort *Mylia verucosa* [41] (Fig. 2D).

Environmental samples were the second most significant source, contributing 18 accessions that yielded novel genomes. Of these, 16 contained novel species (Fig. 2C). Specifically, 14 originated from soil samples, three of which were from metagenomic studies of plant-associated microbiota. Notably, one sample was collected from the sea, and the viral genome is likely the result of contamination (Fig. 2A).

Interestingly, three accessions came from arthropods: the grasshoppers *Locusta migratoria* and *Ceracris nigricornis*, and the phytophagous *Apolygus lucorum* (Fig. 2B). The latter is classified as an isolate of the Aspergillus flavus deltaflexivirus 1. The grasshopper *Ceracris nigricornis* was the only one originating from a single individual (the Bioproject accession refers to an individual: PRJNA543568) (Fig. 2D). The remaining four accessions originated from fungi: two from an edible mushroom *Macrocybe gigantea*, and two from the plant pathogen *Puccinia striiformis* (Fig. 2B).

### Possible new members in the *Tymovirales* order

**Fig. 3:**
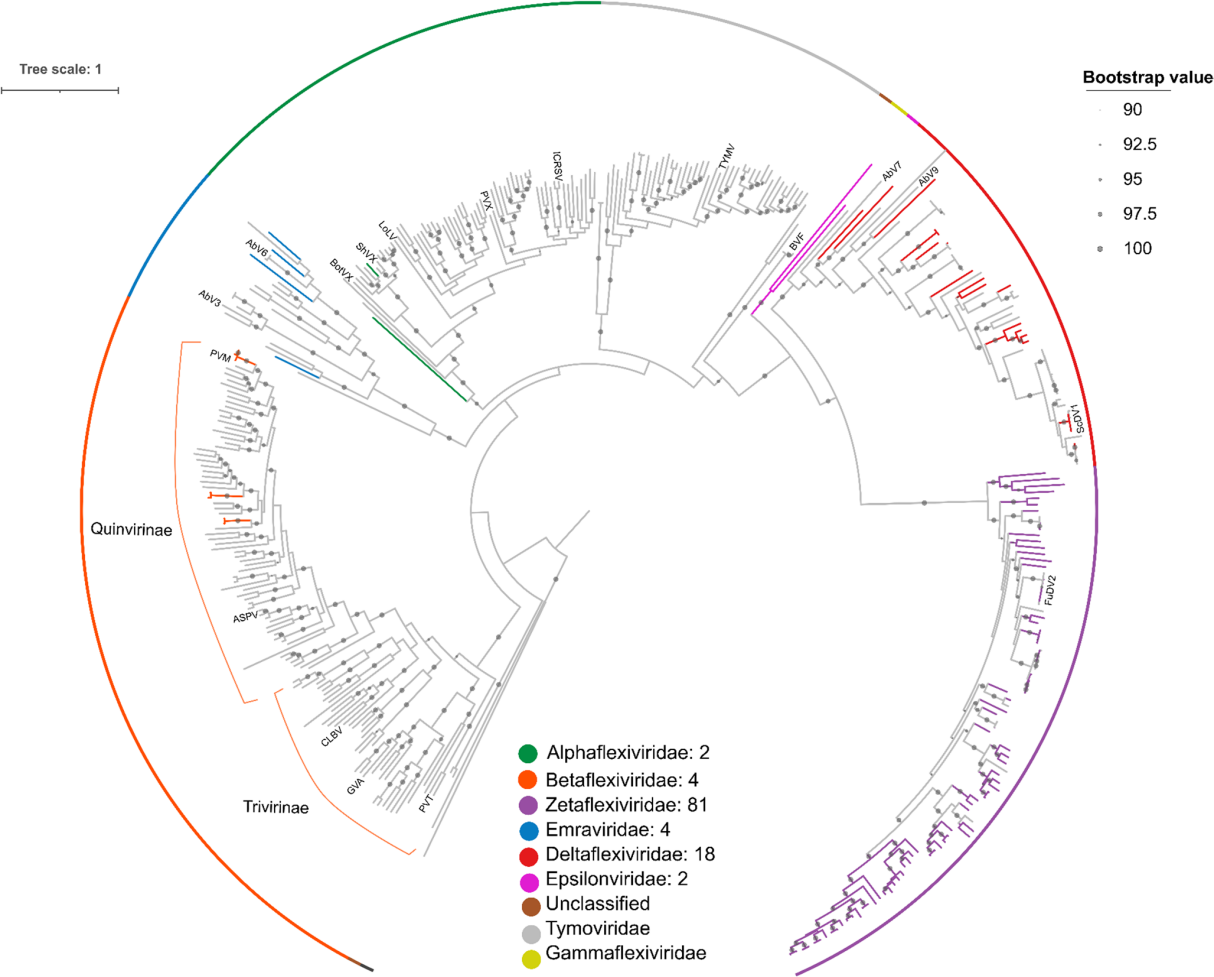
Proposed classification of the *Tymovirales* order. Phylogenetic tree (substitution model: LG + F+R10) of the *Tymovirales* using the whole polyprotein amino acid sequence. The arches delimit the currently recognized and/or proposed families. The branches containing the putative new viruses described in this work are colored according to the assigned family. The legend entry indicates the number of viruses found per family. Species and other taxa of particular interest are included in the tree in text form. The size of the circles indicates the Fast bootstrap value with 10,000 replicates. PVT: potato virus T; GVA: grapevine virus A; ASPV: apple stem pitting virus; PVM: potato virus M; Abv3: Agaricus bisporus virus 3; Abv6: Agaricus bisporus virus; Abv7: Agaricus bisporus virus 7; Abv9: Agaricus bisporus virus 9; BotVX: botrytis virus X; PVX: potato virus X; ICRSV: Indian citrus ringspot virus; TYMV: turnip yellow mosaic virus; BVF: botrytis virus F; ScscDV1: sclerotinia sclerotiorum deltavirus 1; FuDV2: fusarium deltaflexivirus 2

The SRA data analyses using the pipeline yielded 111 possible new viral genomes, listed in Supplementary Table 2. Manual BLASTX searches of predicted ORFs in the new genomes against the nr database revealed similarity hits not only with sequences classified as *Deltaflexiviridae* but also with other members of the *Tymovirales* order. The resulting phylogenetic tree is shown in Fig. 3. The highlighted clades indicate where the mined viral sequences clustered.

The phylogeny of the members of the *Tymovirales* order corroborates the family classification currently accepted for the well-known plant-infecting viruses such as the *Alphaflexiviridae*, *Betaflexiviridae*, and *Tymoviridae*, as they form monophyletic clades (shown in green, orange, and grey in Fig. 3, respectively) with bootstrap values above 90. Additionally, the phylogenetic tree corroborates the subfamily structure within the *Betaflexiviridae* family, with the two subfamilies *Quirivinae* and *Trivinae* forming well-supported clades (shown in orange in Fig. 3). Another well-defined clade, shown in blue in Fig. 3, with high statistical support (bootstrap value above 90), included members that have been suggested to constitute a possible new family [42]. Surprisingly, the clade corresponding to the *Deltaflexiviridae* family, harboring most of the new sequences (100 sequences out of the 111), was split into two large groups (shown in red and purple in Fig. 3). The two clades stemming from a common ancestor displayed considerable divergence from each other. The clear separation between these two clades, as indicated by the length of the purple-marked branch in Fig. 3, suggests that this family can be divided into two families, proposed in this work as *Deltaflexiviridae* and “*Zetaflexiviridae”*. Two other sequences described in this work formed a separate clade (shown in pink in Fig. 3). Given their high divergence from the rest of clade B, these two sequences should also be classified as a new family, proposed in this work as “*Epsilonflexiviridae”*.

**Fig. 4:**
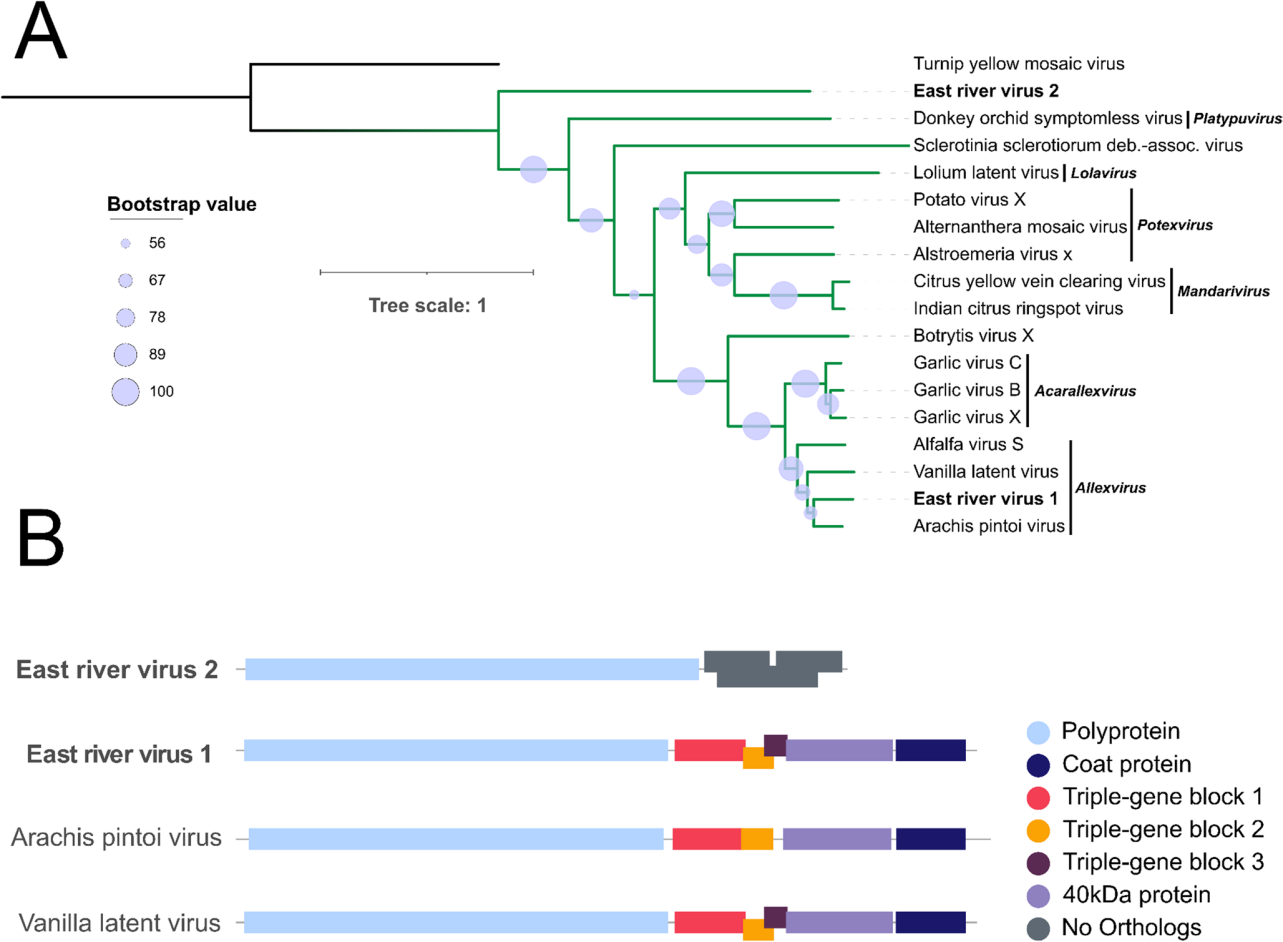
Phylogeny and genomic content of the *Alphaflexiviridae* family. (**A**) Phylogenetic tree (substitution model: LG + F+I+G4) of the *Alphaflexiviridae* family estimated with IQtree using the whole polyprotein amino acid sequence. The size of the circles indicates the Fast bootstrap value with 10,000 replicates. (**B**) Genomic content was drawn with iTOL. Blocks with the same color mark orthologous genes. Genomes described in this work are in bold and italic

Two novel sequences clustered with *Alphaflexiviridae* (marked in green in Fig. 3), and one of them, hereby named East River virus 2, diverged from the common ancestor for the whole family. A second phylogenetic tree, containing a subset of the sequences in Fig. 4, created by sampling three members of each *Alphaflexiviridae* family and using the *Tymoviridae* member turnip yellow mosaic virus as the outgroup, shows that East River virus 2 probably belongs to the *Alphaflexiviridae* family, despite sharing only the polyprotein ORF with the other family members (Fig. 4B). The phylogenetic tree also places the other novel virus, East River virus 1, within the *Allexivirus* genus (Fig. 4A). This grouping is well supported by the high bootstrap value and genomic composition (Fig. 4B), as East River virus 1 shares genes with the other members of the genus. Interestingly, this virus also has a coat protein gene ORF that overlaps with the 40 kDa protein gene ORF, and this ORF is longer than its counterparts.

**Fig. 5:**
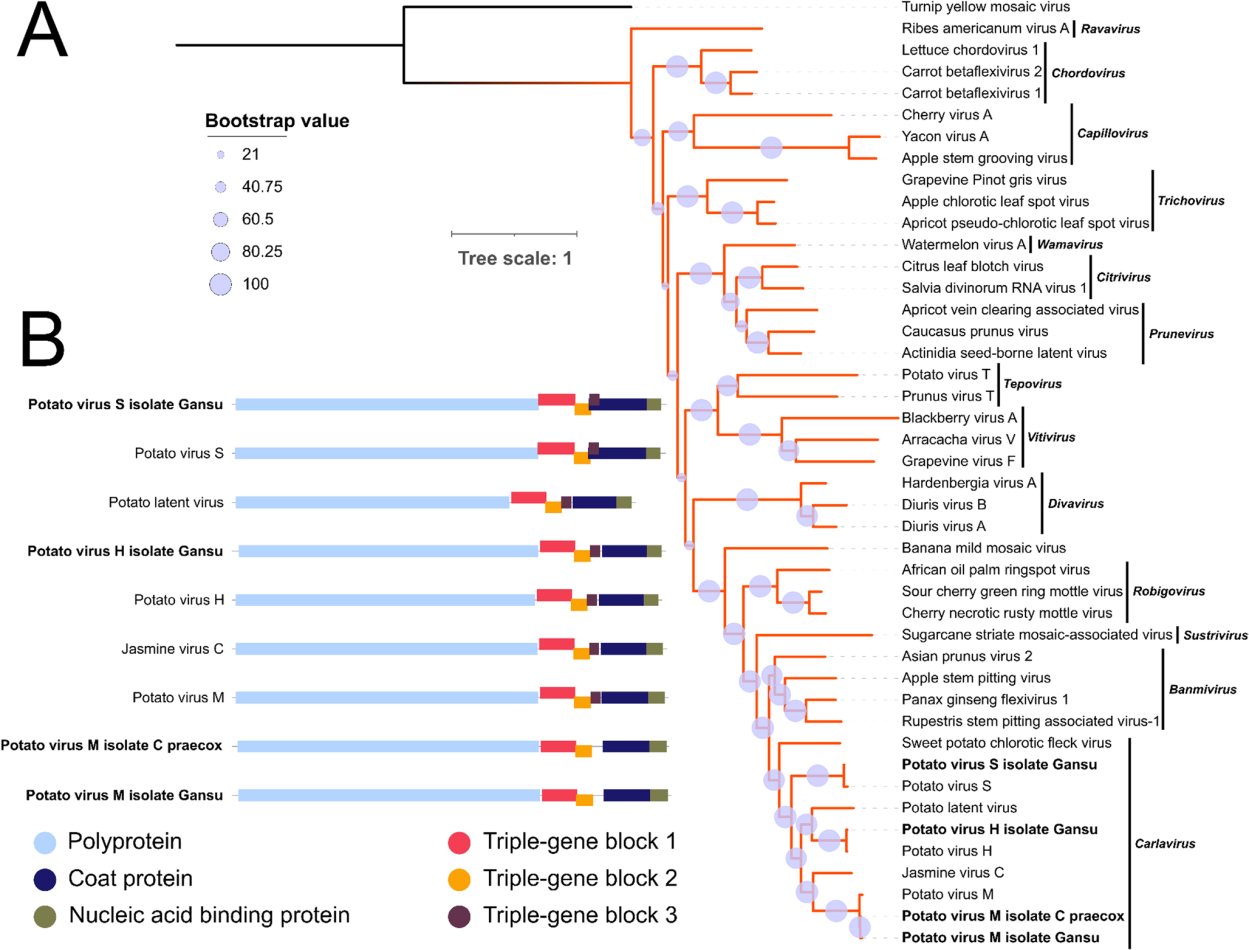
Phylogeny and genomic content of the *Betaflexiviridae* family. (**A**) Phylogenetic tree (substitution model: LG + F+R6) of the *Betaflexiviridae* family estimated with IQtree using the whole polyprotein amino acid sequence. The size of the circles indicates the Fast bootstrap value with 10,000 replicates. The bars indicate the corresponding genus. (**B**) Genomic content drawn with iTOL. Blocks with the same color mark orthologous genes. Genomes described in this work are in bold and italic

The four new sequences grouped with the *Betaflexiviridae* family (Fig. 5). Repeating the same approach used for *Alphaflexiviridae*, three reference sequences were selected from each genus to classify the four newly discovered viruses. The high similarity indicates that these viruses are isolates of the recognized *Carlavirus* species. Three isolates were obtained from samples collected in Gansu. They belonged to the potato virus S (closest BLAST hit identity: AAP76207–98.22%), potato virus H (closest BLAST hit identity: AEI55831–96.1%), and potato virus M (closest BLAST hit identity: YP_277428 − 94.68%). A fourth isolate identified as an isolate of the potato virus M (closest BLAST hit identity: UTQ50775–98.32%), originated from a sample of wintersweet, *Chimonanthus praecox* (Fig. 5).

**Fig. 6:**
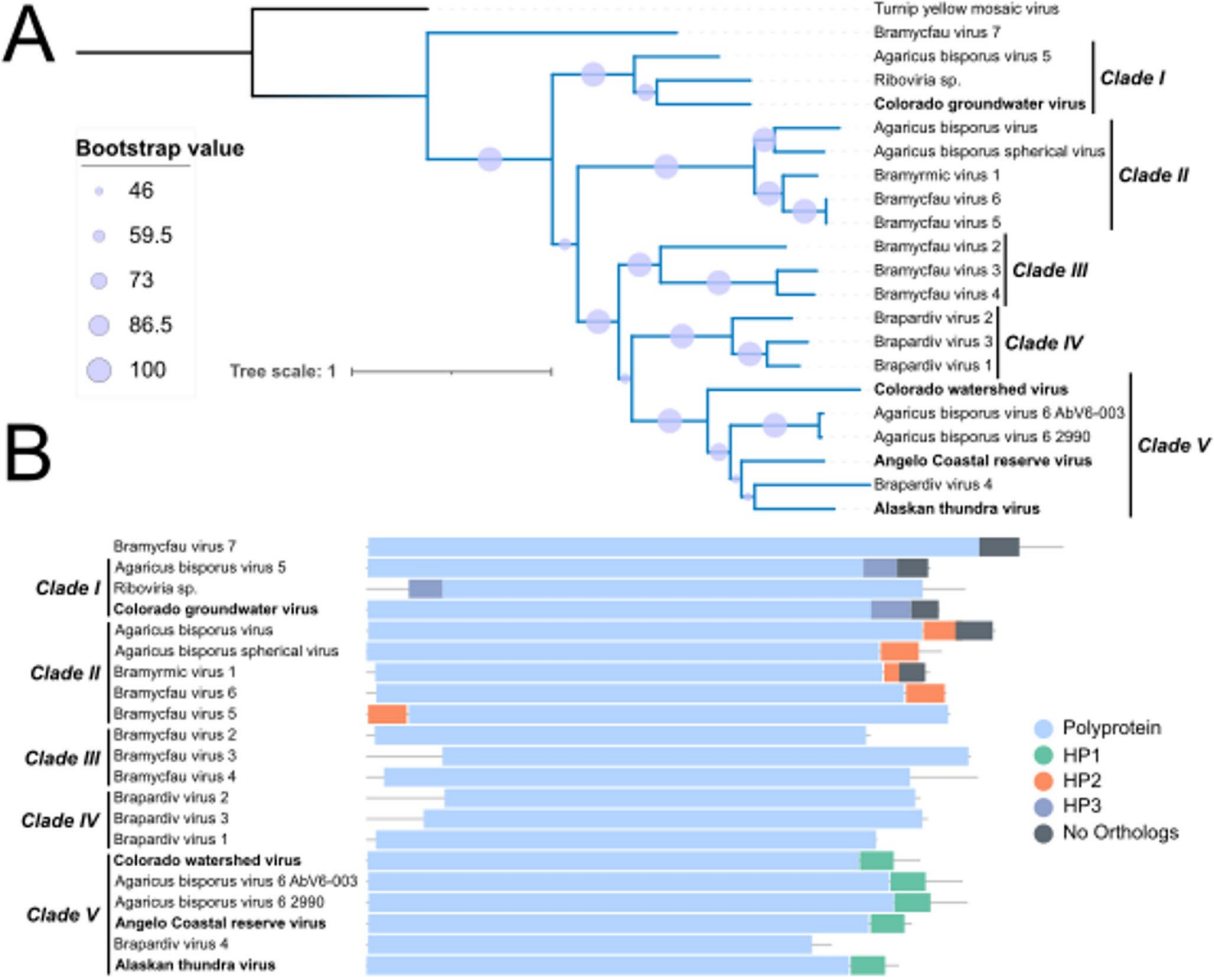
Phylogeny and genomic content of the putative “*Emraviridae”* family. (**A**) Phylogenetic tree (substitution model: LG + F+I+G4) of the family estimated with IQtree using the whole polyprotein amino acid sequence. The size of the circles indicates the Fast bootstrap value with 10,000 replicates. The bars indicate clades that may be assigned as new genera. (**B**) Genomic content drawn with iTOL. Blocks with the same color are orthologous

Four of the new genomes clustered with a distinct group comprising yet-unclassified viruses (shown in blue in Fig. 3), suggesting they can be classified into a new family, provisionally referred to as “*Emraviridae*” [44]. The phylogenetic tree in Fig. 6, based on fewer sequences, indicates that the four genomes (marked in bold) likely represent new species within that “family”. The highest identity to a BLAST hit among the new sequences is 43.4% (AQM32763).

This family has not yet been recognized by the ICTV, thus, there is still no genus-level classification. Yet, there are some indications of a possible genus classification. A combination of orthologous search clustering and phylogenetic analysis revealed five well-defined groups. Three out of the four new viruses clustered with “clade V”, which is also characterized by the presence of HP1 (shown in green in Fig. 6B). The remaining genome clustered within “clade I”.

### *Deltaflexiviridae* family split

**Fig. 7:**
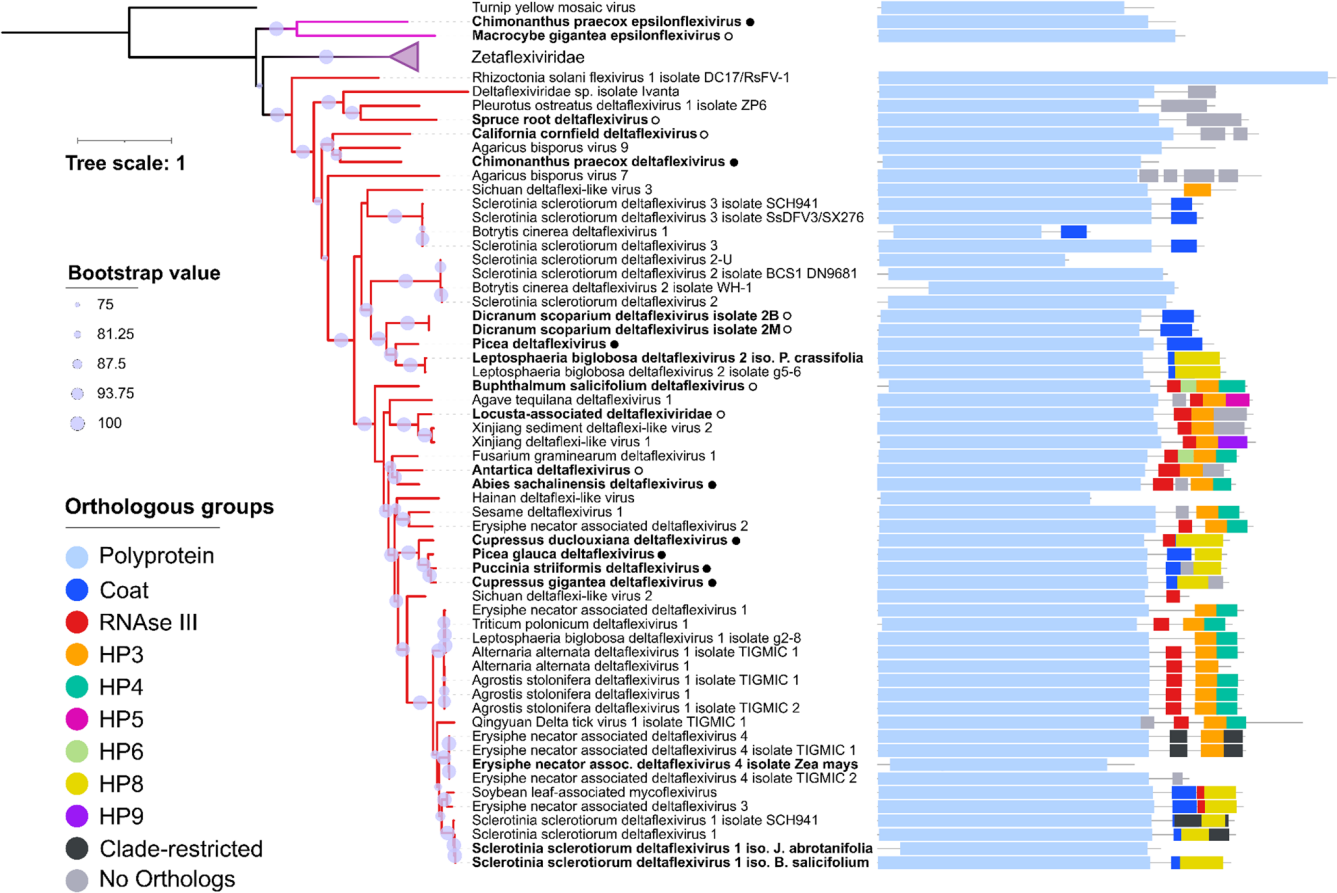
Phylogeny and genomic content of the *Deltaflexiviridae* (in red) and “*Epsilonflexiviridae”* (in pink) families. Phylogenetic tree (substitution model: LG + F+R8) of the *Deltaflexiviridae* family estimated with IQtree using the whole polyprotein amino acid sequence. The size of the circles indicates the Fast bootstrap value with 10,000 replicates. Genomic content drawn with iTOL. Blocks with the same color mark orthologous genes. Genomes described in this work are in bold and italic. White circles mark putative new viruses with polyproteins that share less than 90% identity with known viruses. If these viruses originated from samples sequenced from a single individual, they are additionally marked by black circles

**Fig. 8:**
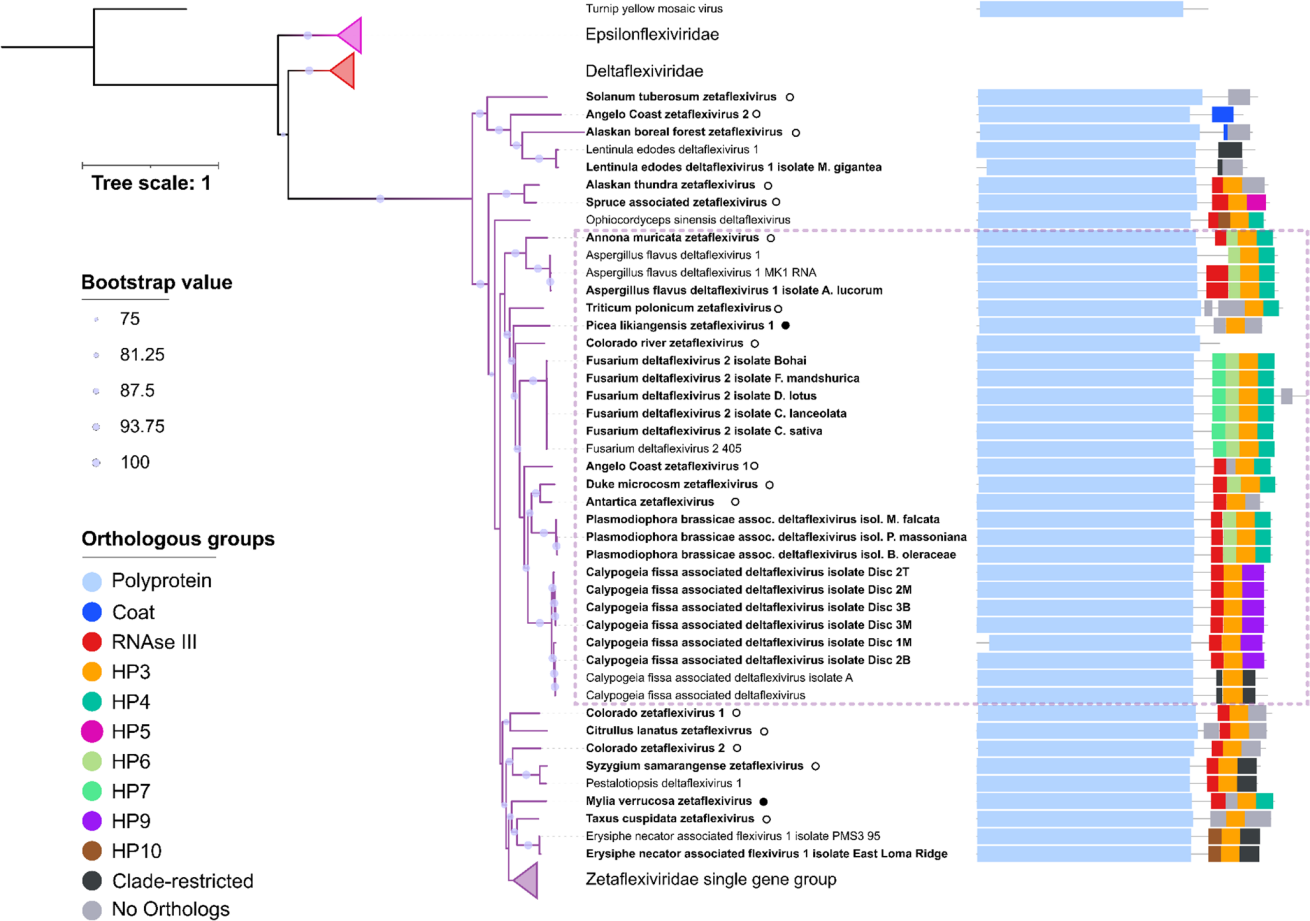
Phylogeny and genomic content of the “*Zetaflexiviridae”* family. Phylogenetic tree (substitution model: LG + F+R8) of the “*Zetaflexiviridae”* family estimated with IQtree using the whole polyprotein amino acid sequence. The size of the circles indicates the Fast bootstrap value with 10,000 replicates. Genomic content was drawn with iTOL. Blocks with the same color are orthologous. The collapsed clade is shown in Fig. 9. The members enclosed within the box are collapsed in Fig. 9. White circles mark putative new viruses with polyproteins that share less than 90% identity with known viruses. If these viruses also originated from samples sequenced from a single individual, they are additionally marked by black circles

Interestingly, most of the sequences described in this work grouped with known members classified in the family *Deltaflexiviridae*. However, the group exhibits a remarkable topology, characterized by three distinct and well-supported clades (Fig. 3). Additionally, the published sequences classified as family members did not form a monophyletic clade. For example, Sclerotinia sclerotiorum deltaflexivirus 1 (Fig. 7), the type species of the family *Deltaflexiviridae*, and Fusarium deltaflexivirus 2 (Fig. 8) are currently classified within the same family, yet they clustered in distinct clades.

**Fig. 9:**
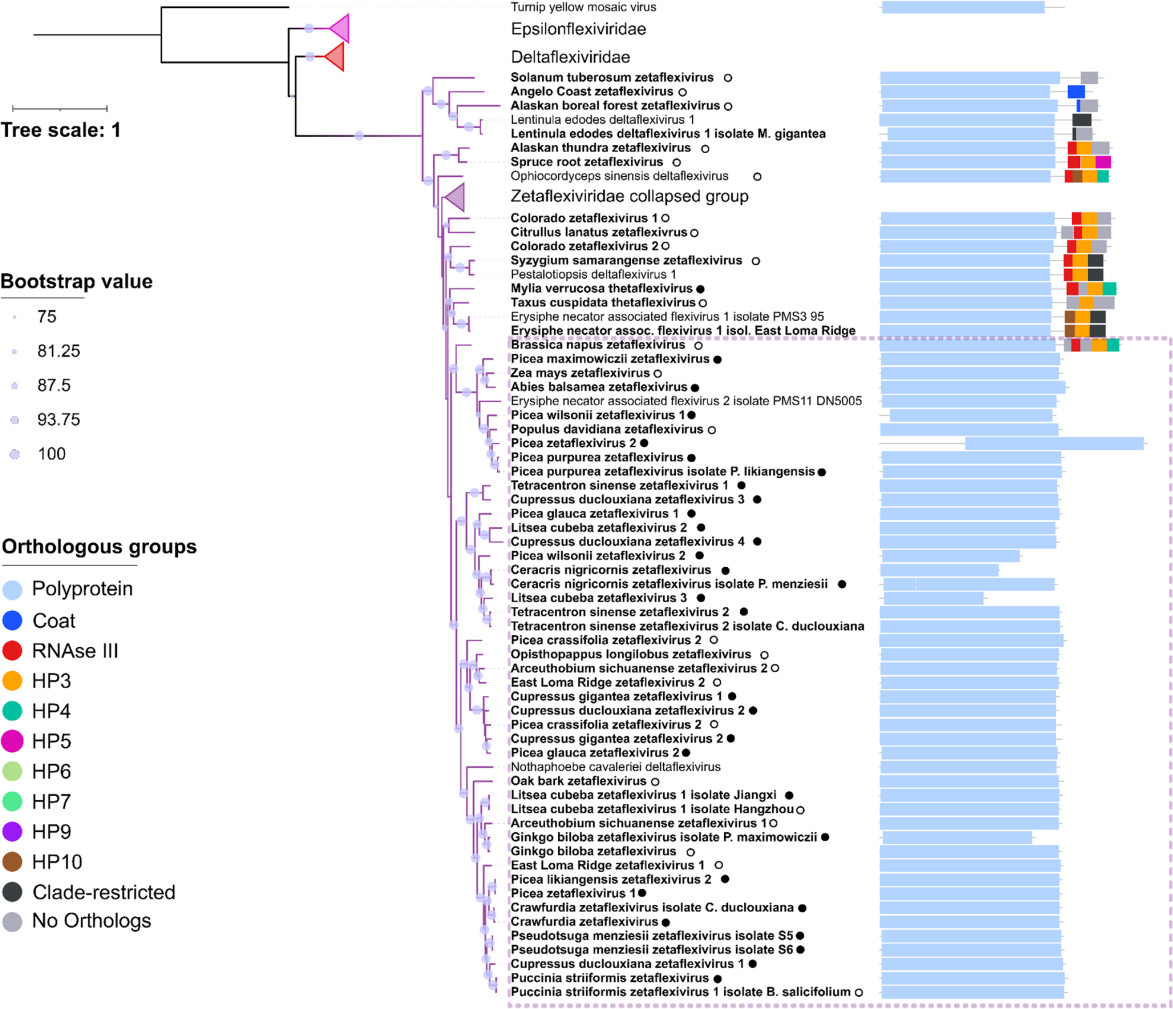
Phylogeny and genomic content of the “*Zetaflexiviridae”* family with the single gene group highlighted. Phylogenetic tree (substitution model: LG + F+R8) of the “*Zetaflexiviridae”* family estimated with IQtree using the whole polyprotein amino acid sequence. The sizes of the circles indicate the Fast bootstrap values with 10,000 replicates. Genomic content was drawn with iTOL. Blocks with the same color are orthologous. The clade comprising members enclosed within the box is collapsed in Fig. 8. White circles mark putative new viruses with polyproteins that share less than 90% identity with known viruses. If these viruses also originated from samples sequenced from a single individual, they are additionally marked by black circles

The high divergence among these three clades suggests they represent different families. Thus, we propose creating two families to more accurately reflect the evolutionary history of this group, as estimated by the phylogenetic analysis: “*Epsilonflexiviridae”* (shown in pink in Fig. 7) and “*Zetaflexiviridae”* (Figs. 8 and 9). The topology of the phylogenetic tree that includes all *Tymovirales* families, and the topologies of the phylogenetic trees focusing on these three families, remain consistent, supporting our proposed classification.

According to the proposed classification, the *Deltaflexiviridae* family will encompass 18 of the 111 newly proposed sequences. The new *Deltaflexiviridae* phylogenetic tree is presented in Fig. 7, with the new genomes shown in bold. Pairwise amino acid identity comparisons revealed that 13 out of the 18 genomes classified into the *Deltaflexiviridae* family belong to new species (marked with circles in Fig. 7, Supplementary Table 2). However, using the more conservative prerequisite and assuming that only genomes mined from samples originating from a single individual were included, 7 of the 18 putative viruses should be classified as new species (marked with black circles in Fig. 7).

The family “*Epsilonflexiviridae”* (depicted in pink in Fig. 7) comprises two members. The amino acid identity between Chimonanthus praecox epsilonflexivirus and the closest sequenced entry in GenBank (QDW81317) is 34.33%. The sample from which this putative virus was derived was from a single wintersweet plant [43], supporting its classification as a new species. In contrast, information regarding the sample of the other family member, *Macrocybe gigantea* epsilonflexivirus, isolated from an edible mushroom, could not be located. Therefore, although it shares 33.83% identity with the closest deposited sequence (QDW81317), the classification of this putative virus as a novel species should be approached with caution.

Meanwhile, the family “*Zetaflexiviridae”* (shown in purple in Fig. 3) harbored 81 of the 111 new sequences. The novel viruses are detailed in Figs. 8 and 9, with a higher-resolution phylogeny shown in bold. To facilitate visualization, portions of the tree were collapsed; specifically, the highlighted section in Fig. 8 is shown collapsed in Fig. 9, and vice versa. Taking the threshold of 90% amino acid identity and the genomic content, 14 novel genomes belong to already recognized species, while 67 novel genomes probably belong to new species (Supplementary Table 2). Taking into account only genomes mined from samples in which a single individual was sequenced, the number drops to 34. Finally, 25 new species meet the single-individual sample criteria within the family *Zetaflexiviridae*.

Interestingly, the genome content is quite diverse within this group. The polyprotein is the only shared gene among all viruses, and seven clusters of orthologs are identified within the group. The five previously described hypothetical proteins (HP2 to HP5) were present in both “*Zetaflexiviridae”* and *Deltaflexiviridae*. Among the orthologs reported in this work, HP6 and HP9 were common to both families, HP8 was exclusive to *Deltaflexiviridae*, and HP7 and HP9 were only detected in *Zetaflexiviridae*. Finally, a notable large clade contains genomes with a single ORF encoding the polyprotein (see the highlighted section in Fig. 9).

## Discussion

Our results confirm the power of virus mining to provide insight into the complex landscape of viral diversity, potentially leading to improved taxonomic classifications. For instance, “*Emraviridae”*, currently an unclassified family [42], was corroborated by the phylogenetic reconstruction, which included our sequences and other similar genomes (Fig. 6). The sequences described in our work clustered with genomes unofficially proposed for this family, Agaricus bisporus virus 5 and Agaricus bisporus virus 7. However, the creation of another family, “*Teagaviridae”*, unofficially proposed in the same work [42], was not supported by our analyses. Indeed, our phylogenetic reconstruction placed the viruses classified in “*Teagaviridae”* (Agaricus bisporus virus 7 and Agaricus bisporus virus 9) within the *Deltaflexiviridae* family (Fig. 7).

Additionally, the newly described viruses indicate the need to create two new families. The family *Epsilonflexiviridae*, named after the fifth letter of the Ancient Greek alphabet (ε, epsilon), and flexi, which refers to the fifth family created from the division of the family previously proposed as *Flexiviridae* [44]. Similarly, the family *Zetaflexiviridae*, named after the sixth letter of the Greek alphabet (ζ, zeta), is the sixth family to be created by dividing the former *Flexiviridae* family. A previous report had suggested that proposing of a new family would better explain the evolutionary history of the family *Deltaflexiviridae* [45].

There is experimental evidence that the Sclerotinia sclerotiorum deltaflexivirus 1 (ScDV1 in Fig. 3) forms a viral particle [23]. Additionally, there is also evidence for horizontal transmission of members in this family [46]. A gene annotated as a coat protein has orthologs present in members of the *Deltaflexiviridae* (blue squares in Fig. 7) and the “*Zetaflexiviridae”* (blue squares in Figs. 8 and 9). Meanwhile, the gene annotated as HP3 shows a distribution complementary to that of the coat protein gene. It is unclear whether HP3 codes for a coat protein, and this is a potential avenue for future research.

A well-known weakness of virus mining in public databases is the difficulty in establishing host-virus relationships, although inferences can be made in that regard [47]. Members of the “*Emraviridae”* may infect only fungi, since all the viruses in this group have been isolated from fungi, and the viruses described in this work originated from environmental samples [42]. Although it is not possible to ascertain the hosts of the viruses from the *Deltaflexiviridae* and “*Zetaflexiviridae”*, there is evidence pointing to both fungi [23, 46] and plants [5], albeit the latter was inferred indirectly.

The completeness of the genome sequences is also a point of debate for viruses mined from public databases [48]. The mined genomes contain no truncated ORFs, and their genome composition is consistent with that of the closest known viruses. The exception is an isolate of Sclerotinia sclerotiorum deltaflexivirus 1 mined from Jacobaea abrotanifolia, which lacks a part of the expected genome (Fig. 7). Additionally, the genomes have the expected size compared to their closest known relatives, except for a few genomes within the single-gene clade (Fig. 9). Still, given the considerable number of genomes that appear complete, there is significant support for our proposal to create two new viral families within the order *Tymovirales*.

Beyond processing obstacles, uncertainty about data quality can also impede progress [12, 49]. The lack of metadata for SRA accessions precludes many conclusions about virus ecology. Interestingly, some annotations include the exact collection date and site. Only 36 of the curated viruses in this study had collection date information. Another potential obstacle is the classification of the discovered viruses. Standard requirements usually involve wet-lab experiments, but samples are often unavailable for such investigations [49]. However, collaborative efforts are underway to initiate discussions on the direction of taxonomic classification in the post-genomic era [48, 50].

This work aimed to elucidate the diversity within the *Deltaflexiviridae* family. A striking diversity, both in evolutionary history and genomic content, was observed, suggesting the creation of two new families and 33 new species. The members of the proposed new species showed less than 90% identity to any other polyprotein sequence and were identified in samples from single individuals (Supplementary Table 2). Our results underscore the importance of large-scale viral mining in public databases. Focusing on specific taxonomic groups or environmental settings can enhance our understanding and characterization of viral diversity.

## Supplementary Information

Below is the link to the supplementary material.

Supplementary Material 1

## Data Availability

The datasets and scripts used in this work are available upon reasonable request from the corresponding author. The sequences are deposited in the Genbank database with accessions BK075158 to BK075268. The accession number of each sequence is detailed in the Supplementary Table 2.
