# Supplementary material for: Mining viruses in public databases unveils the diversity within the Deltaflexiviridae family
Source: Arch Virol. 2026 Jun 26;171(7):223. doi: 10.1007/s00705-026-06648-8 (PMC13309427; doi:10.1007/s00705-026-06648-8)
Supplement: Supplementary file 1 — Supplementary Material 1 [file 705_2026_6648_MOESM1_ESM.docx]

# **Supplementary material**

**Supplementary Table 1: SRA accessions used in this work.** Each accession is identified by its unique Run ID. The information on the sample origin and BioProject was obtained through EUtilities. The information on the samples in which a single individual was sequenced was manually collected. The source article was included when available; otherwise, information was obtained from the sample description on the BioProject webpage.

| Accession | Assembler | BioProject | Sample Origin | Single Individual Sample | Note | Reference |
| --- | --- | --- | --- | --- | --- | --- |
| DRR087270 | SPAdes | PRJDB5527 | Abies sachalinensis | Yes | BioProject refers to the parent individual (No. 236) |  |
| ERR1607812 | SPAdes | PRJEB15280 | Puccinia striiformis | Yes | One lesion per leaf | [[1]](https://www.zotero.org/google-docs/?SV9Mbi) |
| ERR4298181 | SPAdes | PRJEB39098 | Diospyros lotus | No |  |  |
| ERR4298182 | SPAdes | PRJEB39098 | Diospyros lotus | No |  |  |
| ERR4298183 | SPAdes | PRJEB39098 | Diospyros lotus | No |  |  |
| SRR10064007 | SPAdes | PRJNA562115 | Nothaphoebe cavaleriei | Yes | Bioproject refers to individual | [[2]](https://www.zotero.org/google-docs/?5Ievq5) |
| SRR10064028 | SPAdes | PRJNA562115 | Litsea cubeba | Yes | Bioproject refers to individual | [[2]](https://www.zotero.org/google-docs/?d1wiwC) |
| SRR10353554 | SPAdes | PRJNA301093 | Picea asperata |  |  |  |
| SRR10411608 | SPAdes | PRJNA526332 | Apolygus lucorum | Yes | The Illumina library was constructed from a single female adult | [[3]](https://www.zotero.org/google-docs/?mUEEId) |
| SRR10600876 | SPAdes | PRJNA560453 | Cannabis sativa | Yes | Referred to a male leaf |  |
| SRR10600884 | No Assembly | PRJNA560453 | Cannabis sativa | Yes | Referred to a male leaf |  |
| SRR10600885 | SPAdes | PRJNA560453 | Cannabis sativa | Yes | Referred to a male leaf |  |
| SRR10600892 | SPAdes | PRJNA560453 | Cannabis sativa | Yes | Referred to a male leaf |  |
| SRR10799953 | SPAdes | PRJNA596897 | Davidia involucrata |  |  |  |
| SRR10849351 | SPAdes | PRJNA537851 | root metagenome | No | Environmental |  |
| SRR10849399 | No Assembly | PRJNA570392 | soil metagenome | No | Environmental |  |
| SRR10913932 | SPAdes | PRJNA493167 | Taxus cuspidata | No |  |  |
| SRR10919453 | Megahit | PRJNA568306 | soil metagenome | No | Environmental |  |
| SRR10920923 | SPAdes | PRJNA568312 | soil metagenome | No | Environmental |  |
| SRR10968243 | No Assembly | PRJNA603240 | wood decay metagenome |  |  |  |
| SRR11491828 | SPAdes | PRJNA438518 | Homo sapiens |  |  |  |
| SRR11509535 | SPAdes | PRJNA623729 | Davidia involucrata |  |  |  |
| SRR11538445 | SPAdes | PRJNA624360 | Syzygium samarangense | No | Fruits grouped per treatment |  |
| SRR11563839 | SPAdes | PRJNA622045 | soil metagenome | No | Environmental |  |
| SRR11563874 | SPAdes | PRJNA622046 | soil metagenome | No | Environmental |  |
| SRR11565175 | No Assembly | PRJNA622063 | soil metagenome | No | Environmental |  |
| SRR11565434 | SPAdes | PRJNA622071 | soil metagenome | No | Environmental |  |
| SRR11565435 | Megahit | PRJNA622072 | soil metagenome | No | Environmental |  |
| SRR11565436 | Megahit | PRJNA622070 | soil metagenome | No | Environmental |  |
| SRR11565437 | Megahit | PRJNA622074 | soil metagenome | No | Environmental |  |
| SRR11565438 | Megahit | PRJNA622075 | soil metagenome | No | Environmental |  |
| SRR11566795 | Megahit | PRJNA625382 | Tetracentron sinense | Yes | A single individual was sequenced | [[4]](https://www.zotero.org/google-docs/?TSWwg6) |
| SRR11566796 | No Assembly | PRJNA625382 | Tetracentron sinense | Yes | A single individual was sequenced | [[4]](https://www.zotero.org/google-docs/?3yNHOU) |
| SRR11566797 | Megahit | PRJNA625382 | Tetracentron sinense | Yes | A single individual was sequenced | [[4]](https://www.zotero.org/google-docs/?m3DcKX) |
| SRR11613709 | SPAdes | PRJNA625627 | Lens orientalis |  |  |  |
| SRR11614646 | SPAdes | PRJNA628368 | Populus davidiana |  |  |  |
| SRR11614647 | SPAdes | PRJNA628368 | Populus davidiana | No | Unclear |  |
| SRR11614648 | SPAdes | PRJNA628368 | Populus davidiana |  |  |  |
| SRR11947096 | SPAdes | PRJNA600650 | Chimonanthus praecox | Yes | A single individual was sampled | [[5]](https://www.zotero.org/google-docs/?V82bQv) |
| SRR12009638 | SPAdes | PRJNA636634 | Crawfurdia sp. | Yes | The bioproject refer to the specimen voucher (collection) |  |
| SRR12282622 | SPAdes | PRJNA641031 | Pinus massoniana |  |  |  |
| SRR12626868 | SPAdes | PRJNA662998 | Picea crassifolia | No |  |  |
| SRR12632402 | SPAdes | PRJNA661377 | Pestalotiopsis mangiferae |  |  |  |
| SRR12756235 | SPAdes | PRJNA666429 | soil metagenome | No | Environmental |  |
| SRR12756236 | SPAdes | PRJNA666429 | soil metagenome | No | Environmental |  |
| SRR12756237 | SPAdes | PRJNA666429 | soil metagenome | No | Environmental |  |
| SRR12756243 | SPAdes | PRJNA666429 | soil metagenome | No | Environmental |  |
| SRR12756244 | SPAdes | PRJNA666429 | soil metagenome | No | Environmental |  |
| SRR13249293 | SPAdes | PRJNA681878 | Cunninghamia lanceolata | Yes | The seedlings were exposed to the treatments separately | [[6]](https://www.zotero.org/google-docs/?mBENSo) |
| SRR13249295 | SPAdes | PRJNA681878 | Cunninghamia lanceolata | Yes | The seedlings were exposed to the treatments separately | [[6]](https://www.zotero.org/google-docs/?cLpHJk) |
| SRR1562369 | No Assembly |  |  |  |  |  |
| SRR1576647 | SPAdes | PRJNA256347 | Puccinia striiformis | Yes | One lesion per leaf | [[1]](https://www.zotero.org/google-docs/?FwhDMm) |
| SRR1770196 | SPAdes | PRJNA271501 | Locusta migratoria |  |  |  |
| SRR2018870 | SPAdes | PRJNA282959 | rhizosphere metagenome |  |  |  |
| SRR2515940 | SPAdes | PRJNA296922 | Pseudotsuga menziesii | Yes | One tree per sample | [[7]](https://www.zotero.org/google-docs/?eJRAuR) |
| SRR2515951 | SPAdes | PRJNA296922 | Pseudotsuga menziesii | Yes | One tree per sample | [[7]](https://www.zotero.org/google-docs/?3dDsEN) |
| SRR2515952 | No Assembly |  |  |  |  |  |
| SRR2515965 | SPAdes | PRJNA296922 | Pseudotsuga menziesii | Yes | One tree per sample | [[7]](https://www.zotero.org/google-docs/?i8RW42) |
| SRR2515985 | SPAdes | PRJNA296922 | Pseudotsuga menziesii | Yes | One tree per sample | [[7]](https://www.zotero.org/google-docs/?V8Q4KF) |
| SRR2515987 | SPAdes | PRJNA296922 | Pseudotsuga menziesii | Yes | One tree per sample | [[7]](https://www.zotero.org/google-docs/?znGLj1) |
| SRR2515988 | SPAdes | PRJNA296922 | Pseudotsuga menziesii | Yes | One tree per sample | [[7]](https://www.zotero.org/google-docs/?E8pBCq) |
| SRR2515999 | SPAdes | PRJNA296922 | Pseudotsuga menziesii | Yes | One tree per sample | [[7]](https://www.zotero.org/google-docs/?7P9xnA) |
| SRR2905719 | SPAdes | PRJNA301093 | Picea likiangensis | Yes | One tree per sample | [[8]](https://www.zotero.org/google-docs/?YabjeD) |
| SRR2905719 | SPAdes | PRJNA301093 | Picea likiangensis | Yes | One tree per sample | [[8]](https://www.zotero.org/google-docs/?DhwS4r) |
| SRR2905720 | No Assembly |  |  |  |  |  |
| SRR3085214 | SPAdes | PRJNA307530 | Arceuthobium sichuanense | No |  |  |
| SRR4333202 | SPAdes | PRJNA345072 | Brassica oleracea | No |  |  |
| SRR444018 | No Assembly |  |  |  |  |  |
| SRR5127315 | SPAdes | PRJNA357112 | Abies balsamea | Yes | Selected individuals sequenced | [[9]](https://www.zotero.org/google-docs/?El5TY5) |
| SRR5138297 | SPAdes | PRJNA359583 | Macrocybe gigantea |  |  |  |
| SRR5138298 | SPAdes | PRJNA359583 | Macrocybe gigantea |  |  |  |
| SRR5215303 | SPAdes | PRJNA364865 | soil metagenome | No | Enviromental |  |
| SRR5215307 | Megahit | PRJNA364767 | soil metagenome | No | Enviromental |  |
| SRR5237193 | SPAdes | PRJNA371565 | Tragopogon porrifolius |  |  |  |
| SRR5237203 | SPAdes | PRJNA371565 | Jacobaea abrotanifolia | No | Unclear | [[10]](https://www.zotero.org/google-docs/?Uo0dEM) |
| SRR5237210 | SPAdes | PRJNA371565 | Buphthalmum salicifolium | No | Unclear | [[10]](https://www.zotero.org/google-docs/?ujY4F6) |
| SRR5237235 | SPAdes | PRJNA371565 | Achillea ptarmica |  |  |  |
| SRR5252270 | Megahit | PRJNA366856 | soil metagenome | No | Environmental |  |
| SRR5252281 | Megahit | PRJNA366858 | soil metagenome | No | Environmental |  |
| SRR5252282 | Megahit | PRJNA366860 | soil metagenome | No | Environmental |  |
| SRR5252284 | Megahit | PRJNA366861 | soil metagenome | No | Environmental |  |
| SRR5252346 | Megahit | PRJNA366866 | soil metagenome | No | Environmental |  |
| SRR5252349 | Megahit | PRJNA366863 | soil metagenome | No | Environmental |  |
| SRR5252350 | Megahit | PRJNA366865 | soil metagenome | No | Environmental |  |
| SRR5252351 | Megahit | PRJNA366869 | soil metagenome | No | Environmental |  |
| SRR5252352 | Megahit | PRJNA366862 | soil metagenome | No | Environmental |  |
| SRR5252353 | Megahit | PRJNA366868 | soil metagenome | No | Environmental |  |
| SRR5252354 | Megahit | PRJNA366867 | soil metagenome | No | Environmental |  |
| SRR5252356 | Megahit | PRJNA366870 | soil metagenome | No | Environmental |  |
| SRR5252363 | Megahit | PRJNA366871 | soil metagenome | No | Environmental |  |
| SRR5252566 | Megahit | PRJNA366874 | soil metagenome | No | Environmental |  |
| SRR5252567 | Megahit | PRJNA366872 | soil metagenome | No | Environmental |  |
| SRR5252568 | Megahit | PRJNA366877 | soil metagenome | No | Environmental |  |
| SRR5252571 | Megahit | PRJNA366878 | soil metagenome | No | Environmental |  |
| SRR5252572 | Megahit | PRJNA366875 | soil metagenome | No | Environmental |  |
| SRR5252623 | Megahit | PRJNA366879 | soil metagenome | No | Environmental |  |
| SRR5256983 | Megahit | PRJNA365987 | soil metagenome | No | Environmental |  |
| SRR5257112 | Megahit | PRJNA365992 | soil metagenome | No | Environmental |  |
| SRR5257968 | Megahit | PRJNA365988 | soil metagenome | No | Environmental |  |
| SRR5257969 | Megahit | PRJNA365990 | soil metagenome | No | Environmental |  |
| SRR5257970 | Megahit | PRJNA365991 | soil metagenome | No | Environmental |  |
| SRR5257971 | Megahit | PRJNA365989 | soil metagenome | No | Environmental |  |
| SRR5258557 | Megahit | PRJNA365997 | soil metagenome | No | Environmental |  |
| SRR5258558 | Megahit | PRJNA365993 | soil metagenome | No | Environmental |  |
| SRR5258559 | Megahit | PRJNA365996 | soil metagenome | No | Environmental |  |
| SRR5258560 | Megahit | PRJNA365995 | soil metagenome | No | Environmental |  |
| SRR5258561 | No Assembly | PRJNA365994 | soil metagenome | No | Environmental |  |
| SRR5258562 | Megahit | PRJNA365998 | soil metagenome | No | Environmental |  |
| SRR5258656 | Megahit | PRJNA366004 | soil metagenome | No | Environmental |  |
| SRR5258657 | Megahit | PRJNA366002 | soil metagenome | No | Environmental |  |
| SRR5258658 | Megahit | PRJNA366005 | soil metagenome | No | Environmental |  |
| SRR5258659 | Megahit | PRJNA366003 | soil metagenome | No | Environmental |  |
| SRR5258660 | Megahit | PRJNA366007 | soil metagenome | No | Environmental |  |
| SRR5258661 | Megahit | PRJNA366006 | soil metagenome | No | Environmental |  |
| SRR5260218 | Megahit | PRJNA366001 | soil metagenome | No | Environmental |  |
| SRR5260219 | Megahit | PRJNA366009 | soil metagenome | No | Environmental |  |
| SRR5260220 | Megahit | PRJNA366008 | soil metagenome | No | Environmental |  |
| SRR5457454 | SPAdes | PRJNA367202 | freshwater metagenome | No | Environmental |  |
| SRR5468102 | SPAdes | PRJNA365471 | soil metagenome | No | Environmental |  |
| SRR5468103 | SPAdes | PRJNA365471 | soil metagenome | No | Environmental |  |
| SRR5712152 | SPAdes | PRJNA389660 | Litsea cubeba | No | Unclear |  |
| SRR5807703 | SPAdes | PRJNA393070 | Citrus reticulata |  |  |  |
| SRR5807742 | SPAdes | PRJNA393070 | Citrus reticulata |  |  |  |
| SRR5807748 | SPAdes | PRJNA392950 | Picea | Yes | One transcriptome for each tree | [[11]](https://www.zotero.org/google-docs/?3f6oxj) |
| SRR5807755 | SPAdes | PRJNA392950 | Picea | Yes | One transcriptome for each tree | [[11]](https://www.zotero.org/google-docs/?MuEoJz) |
| SRR5807761 | SPAdes | PRJNA392950 | Picea | Yes | One transcriptome for each tree | [[11]](https://www.zotero.org/google-docs/?wf9Gv7) |
| SRR5819003 | SPAdes | PRJNA449266 | soil metagenome | No | Environmental |  |
| SRR5885455 | SPAdes | PRJNA394926 | Brassica napus |  |  |  |
| SRR6011665 | No Assembly |  |  |  |  |  |
| SRR6023863 | SPAdes | PRJNA401149 | Picea wilsonii | Yes | One tree per sample | [[12]](https://www.zotero.org/google-docs/?G4jT7R) |
| SRR6023865 | SPAdes | PRJNA401149 | Picea wilsonii | Yes | One tree per sample | [[12]](https://www.zotero.org/google-docs/?05gqYr) |
| SRR6023899 | SPAdes | PRJNA401149 | Picea purpurea | Yes | One tree per sample | [[12]](https://www.zotero.org/google-docs/?poJXRe) |
| SRR6023928 | SPAdes | PRJNA401149 | Picea likiangensis | Yes | One tree per sample | [[12]](https://www.zotero.org/google-docs/?JYTOe4) |
| SRR6125581 | SPAdes | PRJNA411881 | Ginkgo biloba | No | 20 males harvested | [[13]](https://www.zotero.org/google-docs/?MPl5wf) |
| SRR6223543 | Megahit | PRJNA366857 | soil metagenome | No | Environmental |  |
| SRR6223544 | Megahit | PRJNA366876 | soil metagenome | No | Environmental |  |
| SRR6227499 | Megahit | PRJNA405706 | soil metagenome | No | Environmental |  |
| SRR6288023 | SPAdes | PRJNA418247 | Melampsora larici-populina |  |  |  |
| SRR6371231 | Megahit | PRJNA421095 | soil crust metagenome | No | Environmental |  |
| SRR6371231 | Megahit | PRJNA421095 | soil crust metagenome | No | Environmental |  |
| SRR6371233 | SPAdes | PRJNA421095 | soil crust metagenome | No | Environmental |  |
| SRR6399680 | No Assembly | PRJNA421162 | air metagenome | No |  |  |
| SRR6451340 | SPAdes | PRJNA429127 | Smilax rotundifolia |  |  |  |
| SRR6831301 | SPAdes | PRJNA436826 | Cupressus gigantea |  |  |  |
| SRR6831302 | SPAdes | PRJNA436826 | Cupressus gigantea | Yes | Leaves collected from single individuals | [[14]](https://www.zotero.org/google-docs/?583Tah) |
| SRR6831303 | SPAdes | PRJNA436826 | Cupressus gigantea | Yes | Leaves collected from single individuals | [[14]](https://www.zotero.org/google-docs/?4KSQVf) |
| SRR6841543 | SPAdes | PRJNA436827 | Cupressus duclouxiana | Yes | Leaves collected from single individuals | [[14]](https://www.zotero.org/google-docs/?ceopDp) |
| SRR6841546 | SPAdes | PRJNA436827 | Cupressus duclouxiana | Yes | Leaves collected from single individuals | [[14]](https://www.zotero.org/google-docs/?phOyWz) |
| SRR6841569 | SPAdes | PRJNA436827 | Cupressus duclouxiana | Yes | Leaves collected from single individuals | [[14]](https://www.zotero.org/google-docs/?5UsIFG) |
| SRR6973844 | SPAdes | PRJNA444731 | freshwater sediment | No |  |  |
| SRR6998483 | No Assembly | PRJNA272922 | phyllosphere |  |  |  |
| SRR6998922 | SPAdes | PRJNA432042 | Theridiosoma gemmosum |  |  |  |
| SRR7007636 | Megahit | PRJNA444237 | freshwater sediment | No | Environmental |  |
| SRR7080220 | SPAdes | PRJNA441380 | freshwater metagenome | No | Environmental |  |
| SRR7142645 | SPAdes | PRJNA465238 | soil metagenome | No | Environmental |  |
| SRR7156500 | SPAdes | PRJNA471103 | Opisthopappus longilobus | No |  |  |
| SRR7276853 | SPAdes | PRJNA475012 | plant metagenome | No | Environmental |  |
| SRR7589723 | SPAdes | PRJNA468272 | root metagenome | No | Environmental |  |
| SRR7760051 | SPAdes | PRJNA488137 | Citrullus lanatus | No |  |  |
| SRR7812054 | SPAdes | PRJNA489765 | Malus domestica |  |  |  |
| SRR7945823 | SPAdes | PRJNA493975 | marine metagenome | No | Environmental |  |
| SRR7945827 | SPAdes | PRJNA493975 | marine metagenome | No | Environmental |  |
| SRR7945833 | SPAdes | PRJNA493975 | marine metagenome | No | Environmental |  |
| SRR8145840 | SPAdes | PRJNA499105 | Dicranum scoparium | No |  |  |
| SRR8145842 | SPAdes | PRJNA499105 | Dicranum scoparium | No |  |  |
| SRR8145843 | SPAdes | PRJNA499105 | Dicranum scoparium |  |  |  |
| SRR8145844 | SPAdes | PRJNA499105 | Dicranum scoparium | No |  |  |
| SRR8145845 | SPAdes | PRJNA499105 | Dicranum scoparium | No |  |  |
| SRR8145846 | SPAdes | PRJNA499105 | Dicranum scoparium | No |  |  |
| SRR8145847 | SPAdes | PRJNA499105 | Dicranum scoparium |  |  |  |
| SRR8145848 | SPAdes | PRJNA499105 | Dicranum scoparium | No |  |  |
| SRR8202177 | SPAdes | PRJNA505755 | Mylia verrucosa | Yes | individual shoots and branches | [[15]](https://www.zotero.org/google-docs/?Honc1P) |
| SRR8258355 | SPAdes | PRJNA507459 | Ophiocordyceps sinensis |  |  |  |
| SRR8258356 | SPAdes | PRJNA507459 | Ophiocordyceps sinensis | No |  |  |
| SRR8466599 | SPAdes | PRJNA516040 | Camellia sinensis |  |  |  |
| SRR8530892 | SPAdes | PRJNA520970 | Medicago falcata |  |  |  |
| SRR8550084 | SPAdes | PRJNA519105 | leaf litter metagenome |  |  |  |
| SRR8651747 | SPAdes | PRJNA524759 | Viola inconspicua |  |  |  |
| SRR8724437 | SPAdes | PRJNA526921 | Homo sapiens |  |  |  |
| SRR8816578 | SPAdes | PRJNA474576 | Brassica napus | No |  |  |
| SRR8959862 | SPAdes | PRJNA539840 | Annona muricata | No | Seeds were grounded togther |  |
| SRR9001737 | SPAdes | PRJNA530033 | Zea mays subsp. mays | No |  |  |
| SRR9016051 | SPAdes | PRJNA536004 | leaf litter metagenome | No | Environmental |  |
| SRR9016053 | SPAdes | PRJNA536007 | leaf litter metagenome | No | Environmental |  |
| SRR9016252 | SPAdes | PRJNA536012 | leaf litter metagenome | No | Environmental |  |
| SRR9016264 | SPAdes | PRJNA536017 | leaf litter metagenome | No | Environmental |  |
| SRR9030887 | SPAdes | PRJNA537853 | root metagenome | No | Environmental |  |
| SRR9089667 | SPAdes | PRJNA543568 | Ceracris nigricornis | Yes | Bioproject referred to a male number |  |
| SRR9089668 | SPAdes | PRJNA543568 | Ceracris nigricornis | Yes | Bioproject referred to a male number |  |
| SRR9089669 | SPAdes | PRJNA543568 | Ceracris nigricornis | Yes | Bioproject referred to a male number |  |
| SRR9089670 | SPAdes | PRJNA543568 | Ceracris nigricornis | Yes | Bioproject referred to a male number |  |
| SRR9089673 | SPAdes | PRJNA543568 | Ceracris nigricornis | Yes | Bioproject referred to a male number |  |
| SRR9120836 | SPAdes | PRJNA541919 | Solanum tuberosum |  |  |  |
| SRR9128707 | Megahit | PRJNA530081 | Rhododendron rubiginosum |  |  |  |
| SRR9184244 | SPAdes | PRJNA545953 | Triticum polonicum | No |  |  |
| SRR926244 | Megahit | PRJNA210511 | Picea glauca | Yes | Tissues from one single tree | [[16]](https://www.zotero.org/google-docs/?AWoZk7) |
| SRR926248 | SPAdes | PRJNA210511 | Picea glauca | Yes | Tissues from one single tree | [[16]](https://www.zotero.org/google-docs/?kukUVd) |
| SRR9595773 | SPAdes | PRJNA551096 | Picea maximowiczii | Yes | Individuals were sampled | [[17]](https://www.zotero.org/google-docs/?b4eImb) |
| SRR9596336 | SPAdes | PRJNA550459 | Fraxinus mandshurica | No |  |  |
| SRR9596337 | SPAdes | PRJNA550459 | Fraxinus mandshurica | No |  |  |
| SRR9596345 | SPAdes | PRJNA550459 | Fraxinus mandshurica | No |  |  |
| SRR9596346 | SPAdes | PRJNA550459 | Fraxinus mandshurica | No |  |  |
| SRR9645877 | SPAdes | PRJNA552818 | Homo sapiens |  |  |  |
| SRR9866324 | SPAdes | PRJNA557343 | Dendrobium officinale |  |  |  |

**Supplementary Table 2: Viruses mined in this work.** Viruses classified in this work, with their respective proposed family referred to by their Genbank accession number. The Novel species column indicates sequences in which the polyprotein shares less than 90% identity with its closest publicly available BLAST hit. The Single Individual Sample column indicates sequences that were mined from samples in which a single individual was sequenced. Viruses that fulfill both criteria are highlighted in grey. The average sequencing depth was calculated using BBmap.

| Accession | Virus Name | Species Name | Proposed Family | Novel Species – identity | Single individual sample | Run | Average depth of coverage | Assembler  Coverage | Closest BLAST hit | Identity |
| --- | --- | --- | --- | --- | --- | --- | --- | --- | --- | --- |
| BK075210 | East river virus 2 | East river virus 2 | Alphaflexiviridae | Yes | No | SRR7007636 | 41071.0 | No | AZF99041.1 | 30.87 |
| BK075211 | East river virus 1 | East river virus 1 | Alphaflexiviridae | Yes | No | SRR7007636 | 74.3429 | No | QIC35022.1 | 63.31 |
| BK075179 | Potato virus M isolate C_praecox | Carlavirus misolani | Betaflexiviridae | No | Yes | SRR11947096 | 17.849204 | Yes | UTQ50775.1 | 98.32 |
| BK075253 | Potato virus M isolate Gansu | Carlavirus misolani | Betaflexiviridae | No | No | SRR9120836 | 78407.3279 | No | AAP76207.1 | 98.22 |
| BK075254 | Potato virus S isolate Gansu | Carlavirus sigmasolani | Betaflexiviridae | No | No | SRR9120836 | 21261.9286 | No | YP_277428.1 | 94.68 |
| BK075255 | Potato virus H isolate Gansu | Carlavirus chisolani | Betaflexiviridae | No | No | SRR9120836 | 222794.6016 | No | AEI55831.1 | 96.1 |
| BK075174 | Sclerotinia sclerotiorum deltaflexivirus 1 isolate B. Salicifolium | Deltaflexivirus sclerotiniae | Deltaflexiviridae | No | No | SRR5237210 | 58.7287 | No | YP_009508363.1 | 96.85 |
| BK075215 | Sclerotinia sclerotiorum deltaflexivirus 1 isolate J. abrotanifolia | Deltaflexivirus sclerotiniae | Deltaflexiviridae | No | No | SRR5237203 | 53.8311 | No | YP_009508363.1 | 97.02 |
| BK075228 | Leptosphaeria biglobosa deltaflexivirus 1 isolate P. crassifolia | Leptosphaeria biglobosa deltaflexivirus 1 | Deltaflexiviridae | No | No | SRR12626868 | 140.6931 | No | XHA85871.1 | 94.47 |
| BK075268 | Erysiphe necator associated deltaflexivirus 4 isolate Zea mays | Erysiphe necator associated deltaflexivirus 4 | Deltaflexiviridae | No | No | SRR9001737 | 11534.0 | No | UVF62075.1 | 98.91 |
| BK075160 | Abies sachalinensis deltaflexivirus | Deltaflexivirus sachalinensis | Deltaflexiviridae | Yes | Yes | DRR087270 | 633.0957 | No | QYF50206.1 | 57.73 |
| BK075164 | Antartica deltaflexivirus | Antartica deltaflexivirus | Deltaflexiviridae | Yes | No | SRR6371231 | 478.9922 | No | WZH58871.1 | 56.43 |
| BK075173 | Buphthalmum salicifolium deltaflexivirus 1 | Deltaflexivirus buphthalmi | Deltaflexiviridae | Yes | No | SRR5237210 | 25.2592 | No | YP_009268710.1 | 49.48 |
| BK075176 | California cornfield deltaflexivirus | California cornfield deltaflexivirus | Deltaflexiviridae | Yes | No | SRR5215307 | 103.7477 | No | UUW06602.1 | 47.39 |
| BK075181 | Chimonanthus praecox deltaflexivirus | Deltaflexivirus chimonanthi | Deltaflexiviridae | Yes | Yes | SRR11947096 | 96.6799 | No | UUW06602.1 | 42.87 |
| BK075189 | Cupressus duclouxiana deltaflexivirus | Deltaflexivirus ducloxianae | Deltaflexiviridae | Yes | Yes | SRR6841546 | 67578.0 | No | QYF50206.1 | 55.53 |
| BK075193 | Cupressus gigantea deltaflexivirus | Deltaflexivirus giganteae | Deltaflexiviridae | Yes | Yes | SRR6831303 | 12.8964 | No | QYF50206.1 | 54.45 |
| BK075198 | Dicranum scoparium deltaflexivirus isolate 2M | Deltaflexivirus dicrani | Deltaflexiviridae | Yes | No | SRR8145845 | 1575.0151 | No | UYL94495.1 | 50.08 |
| BK075200 | Dicranum scoparium deltaflexivirus isolate 2B | Deltaflexivirus dicrani | Deltaflexiviridae | Yes | No | SRR8145846 | 5147.3604 | No | UYL94495.1 | 50.08 |
| BK075220 | Locusta-associated deltaflexiviridae | Locusta-associated deltaflexiviridae | Deltaflexiviridae | Yes | No | SRR1770196 | 227.7126 | No | WNM95040.1 | 66.23 |
| BK075233 | Picea deltaflexivirus | Deltaflexivirus picei | Deltaflexiviridae | Yes | Yes | SRR5807748 | 373.5593 | No | UYL94495.1 | 58.01 |
| BK075234 | Picea glauca deltaflexivirus | Deltaflexivirus glauci | Deltaflexiviridae | Yes | Yes | SRR926248 | 19.5836 | No | QYF50206.1 | 55.16 |
| BK075251 | Puccinia striiformis deltaflexivirus | Deltaflexivirus puccinii | Deltaflexiviridae | Yes | Yes | SRR1576647 | 56.6204 | No | QYF50206.1 | 55.43 |
| BK075257 | Spruce associated deltaflexivirus | Spruce associated deltaflexivirus | Deltaflexiviridae | Yes | No | SRR10849351 | 6.864611 | Yes | XRB79024.1 | 37.18 |
| BK075161 | Angelo Coastal reserve virus | Angelo Coastal reserve virus | Emraviridae | Yes | No | SRR5819003 | 95.6135 | No | AQM32763.1 | 42.36 |
| BK075170 | Alaskan thundra virus | Alaskan thundra virus | Emraviridae | Yes | No | SRR12756236 | 32.2134 | No | AQM32763.1 | 43.4 |
| BK075184 | Colorado groundwater virus | Colorado groundwater virus | Emraviridae | Yes | No | SRR6973844 | 26776.0 | No | AQM49927.1 | 40.32 |
| BK075265 | Colorado watershed virus | Colorado watershed virus | Emraviridae | Yes | No | SRR11565436 | 15.518841 | Yes | UQS94374.1 | 36.62 |
| BK075180 | Chimonanthus praecox epsilonflexivirus | Epsiloflexivirus chimonanthi | Epsilonflexiviridae | Yes | Yes | SRR11947096 | 62.268226 | Yes | QDW81317.1 | 34.33 |
| BK075222 | Macrocybe gigantea epsilonflexivirus | Macrocybe gigantea epsilonflexivirus | Epsilonflexiviridae | Yes | No | SRR5138297 | 571.127343 | Yes | QDW81317.1 | 33.83 |
| BK075166 | Aspergillus flavus zetaflexivirus 1 isolate A. lucorum | Aspergillus flavus zetaflexivirus 1 | Zetaflexiviridae | No | Yes | SRR10411608 | 53.289235 | Yes | BED98292.1 | 98.54 |
| BK075177 | Fusarium zetaflexivirus 2 isolate C. Sativa | Fusarium zetaflexivirus 2 | Zetaflexiviridae | No | Yes | SRR10600876 | 53.202314 | Yes | BDQ13824.1 | 99.59 |
| BK075196 | Fusarium deltaflexivirus 2 isolate C. Lanceolata | Fusarium deltaflexivirus 2 | Zetaflexiviridae | No | Yes | SRR13249295 | 148.806556 | Yes | BDQ13824.1 | 99.13 |
| BK075197 | Fusarium deltaflexivirus 2 isolate D. lotus | Fusarium deltaflexivirus 2 | Zetaflexiviridae | No | No | ERR4298183 | 324.270913 | Yes | BDQ13824.1 | 99.64 |
| BK075201 | Calypogeia fissa associated zetaflexivirus isolate Disc_3M | Calypogeia fissa associated zetaflexivirus | Zetaflexiviridae | No | No | SRR8145844 | 4567.496643 | Yes | CAI5383915.1 | 92.45 |
| BK075199 | Calypogeia fissa associated deltaflexivirus isolate Disc_2B | Calypogeia fissa associated zetaflexivirus | Zetaflexiviridae | No | No | SRR8145846 | 233.8983 | No | CAI5383915.1 | 98.11 |
| BK075202 | Calypogeia fissa associated deltaflexivirus isolate Disc_1M | Calypogeia fissa associated zetaflexivirus | Zetaflexiviridae | No | No | SRR8145842 | 607.6274 | No | CAI5383915.1 | 96.62 |
| BK075203 | Calypogeia fissa associated deltaflexivirus isolate Disc_2T | Calypogeia fissa associated zetaflexivirus | Zetaflexiviridae | No | No | SRR8145840 | 41.0492 | No | CAI5383915.1 | 93.42 |
| BK075204 | Calypogeia fissa associated deltaflexivirus isolate Disc_3B | Calypogeia fissa associated zetaflexivirus | Zetaflexiviridae | No | No | SRR8145848 | 1429.3052 | No | CAI5383915.1 | 92.5 |
| BK075205 | Calypogeia fissa associated deltaflexivirus isolate Disc_2M | Calypogeia fissa associated zetaflexivirus | Zetaflexiviridae | No | No | SRR8145845 | 2588.8889 | No | CAI5383915.1 | 92.65 |
| BK075207 | Erysiphe necator associated flexivirus 1 isolate East Loma Ridge | Erysiphe necator associated flexivirus 1 | Zetaflexiviridae | No | No | SRR9016053 | 2838.8314 | No | QKN22686.1 | 97.26 |
| BK075213 | Fusarium deltaflexivirus 2 isolate F. Mandshurica | Fusarium deltaflexivirus 2 | Zetaflexiviridae | No | No | SRR9596346 | 1866.6164 | No | BDQ13824.1 | 99.29 |
| BK075221 | Lentinula edodes deltaflexivirus 1 isolate M. gigantea | Lentinula edodes deltaflexivirus 1 | Zetaflexiviridae | No | No | SRR5138298 | 29.366785 | Yes | QOX06047.1 | 94.49 |
| BK075252 | Fusarium deltaflexivirus 2 isolate Bohai | Fusarium deltaflexivirus 2 | Zetaflexiviridae | No | No | SRR7945833 | 313.5848 | No | BDQ13824.1 | 99.44 |
| BK075158 | Abies balsamea zetaflexivirus | Zetaflexivirus balsamea | Zetaflexiviridae | Yes | Yes | SRR5127315 | 39.112286 | Yes | QKN22646.1 | 61.6 |
| BK075159 | Alaskan boreal forest zetaflexivirus | Alaskan boreal forest zetaflexivirus | Zetaflexiviridae | Yes | No | SRR5468102 | 569632.0 | No | QOX06047.1 | 49.11 |
| BK075162 | Angelo Coast zetaflexivirus 2 | Angelo Coast zetaflexivirus 2 | Zetaflexiviridae | Yes | No | SRR5819003 | 32738.0 | No | CAI5383915.1 | 65.31 |
| BK075163 | Annona muricata zetaflexivirus | Zetaflexivirus annonae | Zetaflexiviridae | Yes | No | SRR8959862 | 88.2699 | No | BED98292.1 | 64.33 |
| BK075165 | Antartica zetaflexivirus | Antartica zetaflexivirus | Zetaflexiviridae | Yes | No | SRR6371231 | 569.0215 | No | CAI5383915.1 | 68.84 |
| BK075167 | Arceuthobium sichuanense zetaflexivirus 1 | Zetaflexivirus alphasichuanense | Zetaflexiviridae | Yes | No | SRR3085214 | 7.571059 | Yes | BDQ13824.1 | 53.71 |
| BK075168 | Arceuthobium sichuanense zetaflexivirus 2 | Zetaflexivirus betasichuanense | Zetaflexiviridae | Yes | No | SRR3085214 | 5.250658 | Yes | QKN22686.1 | 57.18 |
| BK075169 | Alaskan thundra zetaflexivirus | Alaskan thundra zetaflexivirus | Zetaflexiviridae | Yes | No | SRR12756237 | 600.5735 | No | QDH90368.1 | 73.06 |
| BK075171 | Brassica napus zetaflexivirus | Zetaflexivirus rapi | Zetaflexiviridae | Yes | No | SRR8816578 | 146.8812 | No | QKN22686.1 | 60.54 |
| BK075172 | Plasmodiophora brassicae associated zetaflexivirus isolate B. Oleraceae | Thetafelxivirus massoniana | Zetaflexiviridae | Yes | No | SRR4333202 | 415.826239 | Yes | CAI5383915.1 | 64.39 |
| BK075175 | Puccinia striiformis zetaflexivirus isolate B. Salicifolium | Zetaflexivirus puccinii | Zetaflexiviridae | Yes | No | SRR5237210 | 75.6593 | No | QKN22686.1 | 54.6 |
| BK075178 | Ceracris nigricornis zetaflexivirus | Zetaflexivirus betamenziesii | Zetaflexiviridae | Yes | Yes | SRR9089668 | 20.4394 | No | QKN22675.1 | 59.15 |
| BK075182 | Citrullus lanatus deltaflexivrus | Zetaflexivirus citrulli | Zetaflexiviridae | Yes | No | SRR7760051 | 177.0729 | No | BDQ13824.1 | 57.14 |
| BK075183 | Crawfurdia deltaflexivirus | Zetaflexivirus crawfurdiae | Zetaflexiviridae | Yes | Yes | SRR12009638 | 37.655963 | Yes | QKN22686.1 | 55.62 |
| BK075185 | Colorado river deltaflexivirus | Colorado river deltaflexivirus | Zetaflexiviridae | Yes | No | SRR6973844 | 81.3252 | No | XNT21052.1 | 67.37 |
| BK075186 | Crawfurdia deltaflexivirus isolate C. Duclouxiana | Zetaflexivirus crawfurdiae | Zetaflexiviridae | Yes | Yes | SRR6841569 | 39.8123 | No | QKN22686.1 | 55.28 |
| BK075187 | Cupressus duclouxiana zetaflexivirus 1 | Zetaflexivirus alphaducloxianae | Zetaflexiviridae | Yes | Yes | SRR6841543 | 528.7697 | No | XBR32758.1 | 54.0 |
| BK075188 | Cupressus duclouxiana zetaflexivirus 2 | Zetaflexivirus betaducloxianae | Zetaflexiviridae | Yes | Yes | SRR6841543 | 36.4568 | No | QKN22686.1 | 57.02 |
| BK075190 | Tetracentron sinense deltaflexivirus 2 isolate C. duclouxiana | Zetaflexivirus betatetracentri | Zetaflexiviridae | Yes | Yes | SRR6841546 | 93.5578 | No | QKN22675.1 | 61.04 |
| BK075191 | Cupressus duclouxiana zetaflexivirus 3 | Zetaflexivirus gammaducloxianae | Zetaflexiviridae | Yes | Yes | SRR6841546 | 67.4552 | No | QKN22675.1 | 55.83 |
| BK075192 | Cupressus duclouxiana zetaflexivirus 4 | Zetaflexivirus deltaducloxianae | Zetaflexiviridae | Yes | Yes | SRR6841546 | 192.0912 | No | QKN22675.1 | 71.94 |
| BK075194 | Cupressus gigantea zetaflexivirus 1 | Zetaflexivirus alphagiganteae | Zetaflexiviridae | Yes | Yes | SRR6831303 | 39.9254 | No | QKN22686.1 | 57.74 |
| BK075195 | Cupressus gigantea zetaflexivirus 2 | Zetaflexivirus betagiganteae | Zetaflexiviridae | Yes | Yes | SRR6831302 | 36.4325 | No | QKN22686.1 | 58.08 |
| BK075206 | Duke microcosm zetaflexivirus | Duke microcosm zetaflexivirus | Zetaflexiviridae | Yes | No | SRR10919453 | 7312.7869 | No | CAI5383915.1 | 66.48 |
| BK075208 | East Loma Ridge zetaflexivirus 2 | East Loma Ridge zetaflexivirus 2 | Zetaflexiviridae | Yes | No | SRR9016264 | 10.37 | No | BDQ13824.1 | 54.31 |
| BK075209 | East Loma Ridge zetaflexivirus 3 | East Loma Ridge zetaflexivirus 3 | Zetaflexiviridae | Yes | No | SRR9016252 | 24.7723 | No | QKN22686.1 | 56.03 |
| BK075212 | Colorado zetaflexivirus 2 | Colorado zetaflexivirus 2 | Zetaflexiviridae | Yes | No | SRR7007636 | 356.1884 | No | XBR32758.1 | 59.88 |
| BK075214 | Ginkgo biloba zetaflexivirus | Zetaflexivirus betamaximowiczii | Zetaflexiviridae | Yes | No | SRR6125581 | 42.0782 | No | QKN22686.1 | 55.0 |
| BK075216 | Litsea cubeba zetaflexivirus 1 isolate Hangzhou | Zetaflexivirus alphalitseae | Zetaflexiviridae | Yes | No | SRR5712152 | 47.2928 | No | QKN22686.1 | 55.4 |
| BK075217 | Litsea cubeba zetaflexivirus 1 isolate Jiangxi | Zetaflexivirus alphalitseae | Zetaflexiviridae | Yes | Yes | SRR10064028 | 38.143574 | Yes | QKN22686.1 | 55.51 |
| BK075218 | Litsea cubeba zetaflexivirus 2 | Zetaflexivirus betalitseae | Zetaflexiviridae | Yes | Yes | SRR10064028 | 64.485492 | Yes | QKN22675.1 | 71.88 |
| BK075219 | Litsea cubeba zetaflexivirus 3 | Zetaflexivirus gammalitseae | Zetaflexiviridae | Yes | Yes | SRR10064028 | 15.859392 | Yes | QKN22675.1 | 62.39 |
| BK075223 | Plasmodiophora brassicae associated deltaflexivirus isolate M. falcata | Zetaflexivirus massoniana | Zetaflexiviridae | Yes | No | SRR8530892 | 76.6251 | No | CAI5383915.1 | 64.43 |
| BK075224 | Mylia verrucosa zetaflexivirus | Zetaflexivirus myliae | Zetaflexiviridae | Yes | Yes | SRR8202177 | 360.3924 | No | QKN22686.1 | 59.28 |
| BK075225 | Nothaphoebe cavaleriei zetaflexivirus | Zetaflexivirus phoebi | Zetaflexiviridae | Yes | Yes | SRR10064007 | 80.792167 | Yes | QKN22686.1 | 55.25 |
| BK075226 | Oak associated zetaflexivirus | Oak associated zetaflexivirus | Zetaflexiviridae | Yes | No | SRR7276853 | 536.0617 | No | QKN22686.1 | 55.03 |
| BK075227 | Opisthopappus longilobus zetaflexivirus | Zetaflexivirus opisthopappi | Zetaflexiviridae | Yes | No | SRR7156500 | 44776.0 | No | QKN22686.1 | 57.98 |
| BK075229 | Picea crassifolia zetaflexivirus 1 | Zetaflexivirus alphacrassifolii | Zetaflexiviridae | Yes | No | SRR12626868 | 6.697362 | Yes | QKN22686.1 | 57.54 |
| BK075230 | Picea crassifolia zetaflexivirus 2 | Zetaflexivirus betacrassifolii | Zetaflexiviridae | Yes | No | SRR12626868 | 15.74598 | Yes | QKN22686.1 | 57.17 |
| BK075231 | Picea zetaflexivirus 1 | Zetaflexivirus alphapicei | Zetaflexiviridae | Yes | Yes | SRR5807755 | 120.7064 | No | QKN22686.1 | 54.5 |
| BK075232 | Picea zetaflexivirus 2 | Zetaflexivirus betapicei | Zetaflexiviridae | Yes | Yes | SRR5807748 | 133.2288 | No | QKN22646.1 | 62.01 |
| BK075235 | Picea glauca zetaflexivirus 1 | Zetaflexivirus alphaglauci | Zetaflexiviridae | Yes | Yes | SRR926248 | 163077.0 | No | QKN22675.1 | 62.87 |
| BK075236 | Picea glauca zetaflexivirus 2 | Zetaflexivirus betaglauci | Zetaflexiviridae | Yes | Yes | SRR926244 | 14.2588 | No | QKN22686.1 | 58.23 |
| BK075237 | Picea likiangensis zetaflexivirus 1 | Thetalfexivirus alphalikiangensis | Zetaflexiviridae | Yes | Yes | SRR2905719 | 16.098939 | Yes | BDQ13824.1 | 58.71 |
| BK075238 | Picea purpurea zetaflexivirus isolate P. likiangensis | Zetaflexivirus purpuri | Zetaflexiviridae | Yes | Yes | SRR2905719 | 21.396286 | Yes | QKN22646.1 | 61.16 |
| BK075239 | Picea likiangensis zetaflexivirus 2 | Thetalfexivirus betalikiangensis | Zetaflexiviridae | Yes | Yes | SRR6023928 | 120.7064 | No | QKN22686.1 | 54.5 |
| BK075240 | Picea maximowiczii deltaflexivirus 1 | Zetaflexivirus alphamaximowiczii | Zetaflexiviridae | Yes | Yes | SRR9595773 | 210.7028 | No | QKN22646.1 | 61.48 |
| BK075241 | Ginkgo biloba zetaflexivirus isolate P. maximowiczii | Zetaflexivirus betamaximowiczii | Zetaflexiviridae | Yes | Yes | SRR9595773 | 24253.0 | No | QKN22686.1 | 55.11 |
| BK075242 | Plasmodiophora brassicae associated zetaflexivirus isolate P. massoniana | Thetafelxivirus massoniana | Zetaflexiviridae | Yes | No | SRR12282622 | 615.4865 | No | CAI5383915.1 | 64.37 |
| BK075243 | Picea purpurea zetaflexivirus | Zetaflexivirus purpuri | Zetaflexiviridae | Yes | Yes | SRR6023899 | 40.9846 | No | QKN22646.1 | 61.31 |
| BK075244 | Picea wilsonii zetaflexivirus 1 | Zetaflexivirus alphawilsonii | Zetaflexiviridae | Yes | Yes | SRR6023865 | 56.5266 | No | QKN22646.1 | 64.27 |
| BK075245 | Picea wilsonii zetaflexivirus 2 | Zetaflexivirus betawilsonii | Zetaflexiviridae | Yes | Yes | SRR6023863 | 68.1591 | No | QKN22675.1 | 61.68 |
| BK075246 | Populus davidiana zetaflexivirus | Zetaflexivirus populi | Zetaflexiviridae | Yes | No | SRR11614647 | 21.874311 | Yes | QKN22646.1 | 61.77 |
| BK075247 | Pseudotsuga menziesii zetaflexivirus isolate S5 | Zetaflexivirus alphamenziesii | Zetaflexiviridae | Yes | Yes | SRR2515985 | 91.305182 | Yes | QKN22686.1 | 54.42 |
| BK075248 | Ceracris nigricornis zetaflexivirus isolate P. menziesii | Zetaflexivirus betamenziesii | Zetaflexiviridae | Yes | Yes | SRR2515987 | 6.292517 | Yes | QKN22675.1 | 61.27 |
| BK075249 | Pseudotsuga zetaflexivirus deltaflexivirus isolate S6 | Zetaflexivirus alphamenziesii | Zetaflexiviridae | Yes | Yes | SRR2515988 | 1061.399616 | Yes | QKN22686.1 | 54.37 |
| BK075250 | Puccinia striiformis zetaflexivirus | Zetaflexivirus puccinii | Zetaflexiviridae | Yes | Yes | ERR1607812 | 56.964431 | Yes | QKN22686.1 | 54.22 |
| BK075256 | Solanum tuberosum zetaflexivirus | Zetaflexivirus solani | Zetaflexiviridae | Yes | No | SRR9120836 | 268.6767 | No | QOX06047.1 | 47.8 |
| BK075258 | Spruce associated zetaflexivirus | Spruce associated zetaflexivirus | Zetaflexiviridae | Yes | No | SRR7589723 | 34.0832 | No | QDH90368.1 | 74.81 |
| BK075259 | Syzygium samarangense zetaflexivirus | Zetaflexivirus samarangense | Zetaflexiviridae | Yes | No | SRR11538445 | 81.217902 | Yes | XBR32758.1 | 86.79 |
| BK075260 | Taxus cuspidata zetaflexivirus | Zetaflexivirus taxi | Zetaflexiviridae | Yes | No | SRR10913932 | 57.093608 | Yes | QKN22686.1 | 63.8 |
| BK075261 | Tetracentron sinense zetaflexivirus 1 | Zetaflexivirus alphatetracentri | Zetaflexiviridae | Yes | Yes | SRR11566797 | 33.1044 | No | QKN22675.1 | 54.87 |
| BK075262 | Tetracentron sinense zetaflexivirus 2 | Zetaflexivirus betatetracentri | Zetaflexiviridae | Yes | Yes | SRR11566795 | 74.400748 | Yes | QKN22675.1 | 61.29 |
| BK075263 | Triticum polonicum zetaflexivirus | Zetaflexivirus triticum | Zetaflexiviridae | Yes | No | SRR9184244 | 185.5814 | No | XNT21052.1 | 59.71 |
| BK075264 | Angelo Coast zetaflexivirus | Angelo Coast zetaflexivirus | Zetaflexiviridae | Yes | No | SRR7142645 | 7056.8577 | No | QOX06047.1 | 53.6 |
| BK075266 | Colorado zetaflexivirus 1 | Colorado zetaflexivirus 1 | Zetaflexiviridae | Yes | No | SRR11565436 | 55.855201 | Yes | QTH80200.1 | 57.55 |
| BK075267 | Zea mays zetaflexivirus | Zetaflexivirus zeae | Zetaflexiviridae | Yes | No | SRR9001737 | 120.4314 | No | QKN22646.1 | 60.47 |

#

# References

[1.](https://www.zotero.org/google-docs/?R1qzUc)  [Hubbard A, Lewis CM, Yoshida K, et al (2015) Field pathogenomics reveals the emergence of a diverse wheat yellow rust population. Genome Biol 16:23. https://doi.org/10.1186/s13059-015-0590-8](https://www.zotero.org/google-docs/?R1qzUc)

[2.](https://www.zotero.org/google-docs/?R1qzUc)  [Chen Y-C, Li Z, Zhao Y-X, et al (2020) The Litsea genome and the evolution of the laurel family. Nat Commun 11:1675. https://doi.org/10.1038/s41467-020-15493-5](https://www.zotero.org/google-docs/?R1qzUc)

[3.](https://www.zotero.org/google-docs/?R1qzUc)  [Liu Y, Liu H, Wang H, et al (2021) Apolygus lucorum genome provides insights into omnivorousness and mesophyll feeding. Mol Ecol Resour 21:287–300. https://doi.org/10.1111/1755-0998.13253](https://www.zotero.org/google-docs/?R1qzUc)

[4.](https://www.zotero.org/google-docs/?R1qzUc)  [Liu P-L, Zhang X, Mao J-F, et al (2020) The Tetracentron genome provides insight into the early evolution of eudicots and the formation of vessel elements. Genome Biol 21:291. https://doi.org/10.1186/s13059-020-02198-7](https://www.zotero.org/google-docs/?R1qzUc)

[5.](https://www.zotero.org/google-docs/?R1qzUc)  [Shang J, Tian J, Cheng H, et al (2020) The chromosome-level wintersweet (Chimonanthus praecox) genome provides insights into floral scent biosynthesis and flowering in winter. Genome Biol 21:200. https://doi.org/10.1186/s13059-020-02088-y](https://www.zotero.org/google-docs/?R1qzUc)

[6.](https://www.zotero.org/google-docs/?R1qzUc)  [Chen W, Zhou M, Zhao M, et al (2021) Transcriptome analysis provides insights into the root response of Chinese fir to phosphorus deficiency. BMC Plant Biol 21:. https://doi.org/10.1186/s12870-021-03245-6](https://www.zotero.org/google-docs/?R1qzUc)

[7.](https://www.zotero.org/google-docs/?R1qzUc)  [Hess M, Wildhagen H, Junker LV, Ensminger I (2016) Transcriptome responses to temperature, water availability and photoperiod are conserved among mature trees of two divergent Douglas-fir provenances from a coastal and an interior habitat. BMC Genomics 17:682. https://doi.org/10.1186/s12864-016-3022-6](https://www.zotero.org/google-docs/?R1qzUc)

[8.](https://www.zotero.org/google-docs/?R1qzUc)  [Ru D, Mao K, Zhang L, et al (2016) Genomic evidence for polyphyletic origins and interlineage gene flow within complex taxa: a case study of Picea brachytyla in the Qinghai-Tibet Plateau. Mol Ecol 25:2373–2386. https://doi.org/10.1111/mec.13656](https://www.zotero.org/google-docs/?R1qzUc)

[9.](https://www.zotero.org/google-docs/?R1qzUc)  [Baker EAG, Wegrzyn JL, Sezen UU, et al (2018) Comparative Transcriptomics Among Four White Pine Species. G3 Bethesda Md 8:1461–1474. https://doi.org/10.1534/g3.118.200257](https://www.zotero.org/google-docs/?R1qzUc)

[10.](https://www.zotero.org/google-docs/?R1qzUc)  [Jayasena AS, Fisher MF, Panero JL, et al (2017) Stepwise Evolution of a Buried Inhibitor Peptide over 45 My. Mol Biol Evol 34:1505–1516. https://doi.org/10.1093/molbev/msx104](https://www.zotero.org/google-docs/?R1qzUc)

[11.](https://www.zotero.org/google-docs/?R1qzUc)  [Sun Y, Abbott RJ, Lu Z, et al (2018) Reticulate evolution within a spruce (Picea) species complex revealed by population genomic analysis. Evol Int J Org Evol 72:2669–2681. https://doi.org/10.1111/evo.13624](https://www.zotero.org/google-docs/?R1qzUc)

[12.](https://www.zotero.org/google-docs/?R1qzUc)  [Ru D, Sun Y, Wang D, et al (2018) Population genomic analysis reveals that homoploid hybrid speciation can be a lengthy process. Mol Ecol 27:4875–4887. https://doi.org/10.1111/mec.14909](https://www.zotero.org/google-docs/?R1qzUc)

[13.](https://www.zotero.org/google-docs/?R1qzUc)  [Ni J, Dong L, Jiang Z, et al (2018) Comprehensive transcriptome analysis and flavonoid profiling of Ginkgo leaves reveals flavonoid content alterations in day–night cycles. PLoS ONE 13:e0193897. https://doi.org/10.1371/journal.pone.0193897](https://www.zotero.org/google-docs/?R1qzUc)

[14.](https://www.zotero.org/google-docs/?R1qzUc)  [Ma Y, Wang J, Hu Q, et al (2019) Ancient introgression drives adaptation to cooler and drier mountain habitats in a cypress species complex. Commun Biol 2:213. https://doi.org/10.1038/s42003-019-0445-z](https://www.zotero.org/google-docs/?R1qzUc)

[15.](https://www.zotero.org/google-docs/?R1qzUc)  [Dong S, Zhao C, Zhang S, et al (2019) The Amount of RNA Editing Sites in Liverwort Organellar Genes Is Correlated with GC Content and Nuclear PPR Protein Diversity. Genome Biol Evol 11:3233–3239. https://doi.org/10.1093/gbe/evz232](https://www.zotero.org/google-docs/?R1qzUc)

[16.](https://www.zotero.org/google-docs/?R1qzUc)  [Warren RL, Keeling CI, Yuen MMS, et al (2015) Improved white spruce (Picea glauca) genome assemblies and annotation of large gene families of conifer terpenoid and phenolic defense metabolism. Plant J Cell Mol Biol 83:189–212. https://doi.org/10.1111/tpj.12886](https://www.zotero.org/google-docs/?R1qzUc)

[17.](https://www.zotero.org/google-docs/?R1qzUc)  [Shao C-C, Shen T-T, Jin W-T, et al (2019) Phylotranscriptomics resolves interspecific relationships and indicates multiple historical out-of-North America dispersals through the Bering Land Bridge for the genus Picea (Pinaceae). Mol Phylogenet Evol 141:106610. https://doi.org/10.1016/j.ympev.2019.106610](https://www.zotero.org/google-docs/?R1qzUc)
